# Prolonged fixation and post-mortem delay impede the study of adult neurogenesis in mice

**DOI:** 10.1038/s42003-023-05367-z

**Published:** 2023-09-23

**Authors:** M. Gallardo-Caballero, C. B. Rodríguez-Moreno, L. Álvarez-Méndez, J. Terreros-Roncal, M. Flor-García, E. P. Moreno-Jiménez, A. Rábano, M. Llorens-Martín

**Affiliations:** 1 Department of Molecular Neuropathology, Centro de Biología Molecular “Severo Ochoa” (CBMSO), Spanish Research Council (CSIC)–Universidad Autónoma de Madrid (UAM), Madrid, Spain; 2 Center for Networked Biomedical Research on Neurodegenerative Diseases (CIBERNED), Madrid, Spain; 3 Department of Molecular Biology, Faculty of Sciences, Universidad Autónoma de Madrid, Madrid, Spain; 4 CIEN Foundation, Madrid, Spain

**Keywords:** Adult neurogenesis, Neural stem cells

## Abstract

Adult hippocampal neurogenesis (AHN) gives rise to new neurons throughout life. This phenomenon takes place in more than 120 mammalian species, including humans, yet its occurrence in the latter was questioned after one study proposed the putative absence of neurogenesis markers in the adult human hippocampus. In this regard, we showed that prolonged fixation impedes the visualization of Doublecortin^+^ immature neurons in this structure, whereas other authors have suggested that a dilated post-mortem delay (PMD) underlies these discrepancies. Nevertheless, the individual and/or additive contribution of fixation and the PMD to the detection (or lack thereof) of other AHN markers has not been studied to date. To address this pivotal question, we used a tightly controlled experimental design in mice, which allowed the dissection of the relative contribution of the aforementioned factors to the visualization of markers of individual AHN stages. Fixation time emerged as the most prominent factor globally impeding the study of this process in mice. Moreover, the visualization of other particularly sensitive epitopes was further prevented by prolonged PMD. These results are crucial to disambiguate current controversies related to the occurrence of AHN not only in humans but also in other mammalian species.

## Abstract (web summary)

Significant impact of post-mortem time and fixation method on immunohistochemistry performance of antibody-based staining of common markers relevant to adult neurogenesis.

## Introduction

The mammalian hippocampus is one of the few regions of the brain to host the addition of new neurons during adulthood^1^. As a result of adult hippocampal neurogenesis (AHN), new dentate granule cells (DGCs) are incorporated into classic^2^ and alternative^3^ trisynaptic hippocampal circuits throughout life. Therefore, the addition of new neurons confers enhanced plasticity to the aging mammalian brain. The stages encompassed by AHN have been extensively dissected using immunohistochemical methods, in which specific cell markers are used to visualize cells at each phase of this process^4^. A population of radial-glia-like (RGL) cells with astrocyte-like properties residing in the subgranular zone (SGZ) of the dentate gyrus (DG) sustain the generation of new neurons^5^. These RGL cells orchestrate AHN through interactions with non-neurogenic astrocytes and the DG vasculature^6^. RGL cells, also commonly referred to as neural stem cells (NSCs), are positive for astrocyte markers, such as Nestin, Glial fibrillary acidic protein (GFAP), Vimentin, and SRY (sex determining region Y)-box 2 (Sox2), while they are negative for the mature astrocyte marker S100 calcium-binding protein β (S100β)^7^. Despite being mostly quiescent^8^, adult RGL cells occasionally divide^9^ and give rise to intermediate progenitors, which are mitotically active and express cell proliferation markers, such as Ki67. These cells substantially expand the neurogenic cell population during a limited period^4,10^. After becoming committed to the neuronal lineage, newborn neurons transiently express immaturity markers, such as Doublecortin (DCX)^11–13^, Calretinin (CR), and Polysialylated-neural cell adhesion molecule (PSA-NCAM)^4,14^. Immature DGCs exit the cell cycle and complete their differentiation before becoming fully integrated into the hippocampal circuitry^15^. Throughout their maturation in rodents, these cells receive sequential waves of excitatory and inhibitory innervation^16,17^, progressively increase their dendritic complexity^15^, send their axons towards the CA3 and the CA2 hippocampal subfields^3,15^, and replace the expression of CR by that of Calbindin (CB)^14^.

The occurrence of AHN has been reported in more than 120 mammalian species (reviewed in ref. ^18^). However, contradictory results obtained in particular animal species are also found in the literature^19–21^. In this regard, one of the most disputed points in recent years is the extent to which AHN takes place in the human brain^22,23^. The first report showing the incorporation of systemically administered 5-bromo-2′-deoxyuridine (BrdU) into individual human DGCs dates back to 1998^24^. The occurrence of AHN in humans was further supported by numerous studies that applied a variety of methodologies^25–27^. However, in 2018, contradicting the aforementioned coetaneous studies, the absence of AHN markers in the human brain was affirmed^28^. In this regard, recent data published by our group demonstrated that various technical aspects related to the methodologies used to process human brain samples are crucial to visualize AHN markers in the human brain^23^. We showed that prolonged fixation abolishes the detection of DCX^+^ immature neurons in the adult^29,30^ and infantile^18^ human hippocampus. Similarly, others have reported a negative effect of age and the post-mortem delay (PMD)— defined as the time elapsed between *exitus* and sample immersion in fixative—on the detection of DCX protein in rats^31,32^. However, the extent to which the PMD and fixation protocols affect the visualization of other cell markers related to AHN has not been addressed to date.

Technical, legal, and ethical reasons often prevent the experimental manipulation of the PMD and/or the fixation time in studies involving human samples^33^. Therefore, dissecting the individual and/or additive contributions of these two factors to the detection (or lack thereof) of AHN markers is key to disambiguating controversial aspects related to the study of this phenomenon not only in humans but also in other mammalian species. To tackle these crucial questions, we used a tightly controlled experimental design in mice that allowed us to determine the individual and combined effects of prolonged PMD and fixation time on the study of the distinct stages encompassed by AHN. Our results point to fixation time as the most prominent factor globally impeding the study of AHN in mice. Moreover, we addressed whether the visualization of other particularly sensitive epitopes was further (quantitatively and qualitatively) affected by prolonged PMD intervals.

## Results

### Impact of prolonged fixation and post-mortem delay (PMD) intervals on the visualization of immature neurons in the murine dentate gyrus (DG)

**Fig. 1:**
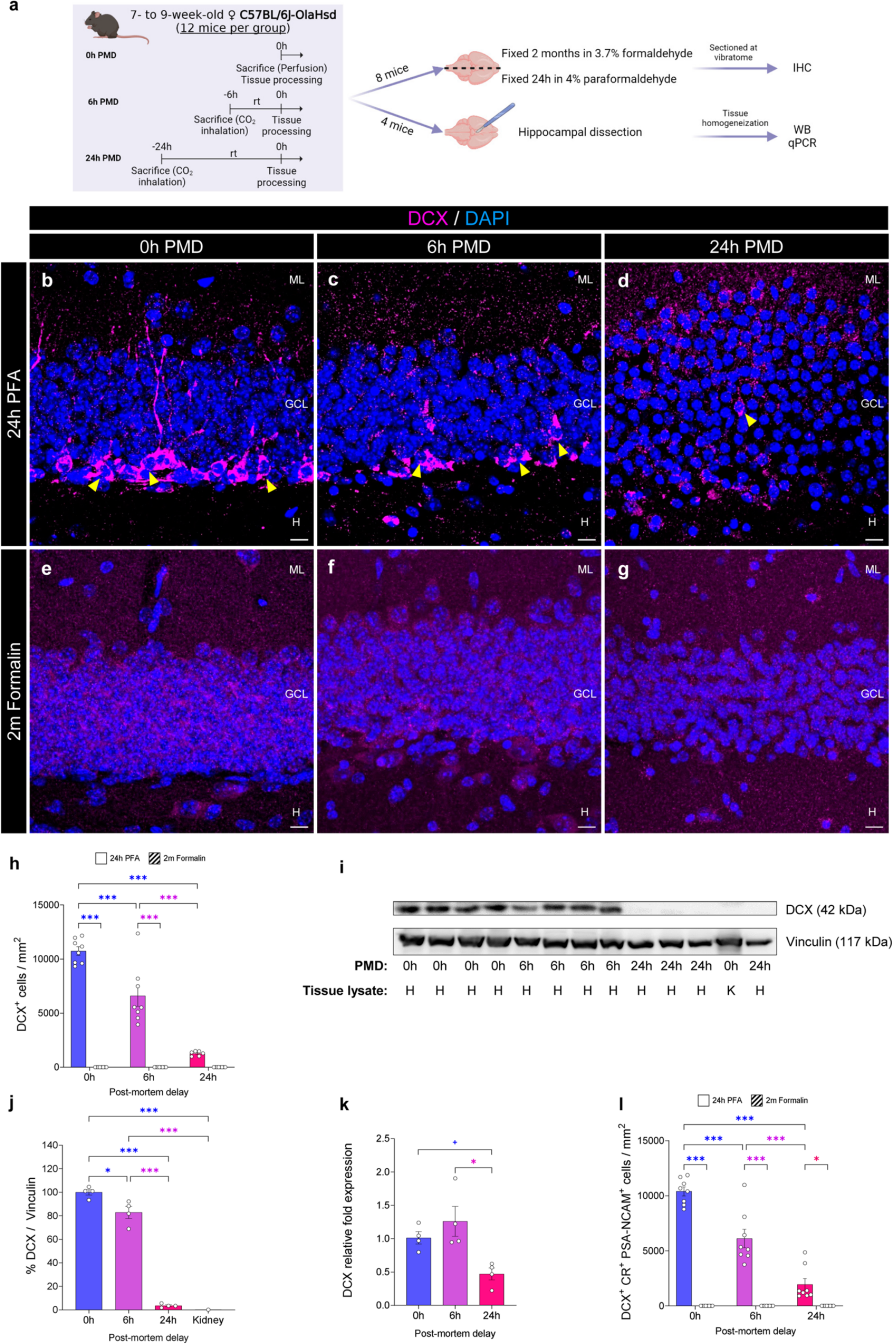
Impact of the post-mortem delay (PMD) and fixation time on the detection of immature dentate granule cells (DGCs). **a** Experimental design. **b**–**g** Representative images of Doublecortin (DCX) staining under distinct experimental conditions. A guinea pig anti-DCX antibody (Synaptic Systems #326004#) was used. **h** Density of DCX^+^ immature DGCs detected with a guinea pig anti-DCX antibody (Synaptic Systems #326004#). **i**, **j** Normalized levels of DCX protein detected by western blot (WB) analysis. **k** Normalized levels of DCX mRNA expression detected by quantitative polymerase chain reaction (qPCR). **l** Density of triple-labeled DCX^+^ Calretinin (CR)^+^ Polysialylated-neural cell adhesion molecule (PSA-NCAM)^+^ immature DGCs detected. In **a**, the illustration was created with BioRender.com. In **b**–**g**, Z-projection images are shown. IHC immunohistochemistry. ML Molecular layer. GCL Granule cell layer. H Hilus. In **i**, H hippocampus, K kidney. White scale bar: 10 μm. Yellow triangles: DCX^+^ immature DGCs. Graphs represent mean values ± SEM. In **h** and **l**: *n* = 8 mice. In **j**, **k** *n* = 4 mice. + 0.1 > *p* ≥ 0.05; *0.05 > *p* ≥ 0.01; and ***p < 0.001.

So-called *ideal conditions* for immunohistochemical studies are characterized by short fixation times and the absence of a PMD interval^18^. However, brain samples of human origin, as well as those obtained from other species living in the wild, are often far from meeting these requirements. Using a strictly controlled mouse experiment, we aimed to dissect the individual and additive contribution of fixation time and PMDs to the immunohistochemical detection of distinct AHN markers. We first studied the performance of various antibodies widely used to label immature DGCs (Fig. 1, Supplementary Figs. 1–5, and Supplementary Table 1), such as DCX, a marker of immature neurons and neuroblasts in the adult mammalian brain^10^. We compared the specificity and qualitative aspects of the signal obtained with 10 distinct commercial anti-DCX antibodies (Supplementary Figs. 2–5) in samples subjected to PMD and fixation of variable durations. The signal quality obtained with these antibodies showed slight variability even under *ideal* conditions (namely, 24 h of fixation at 4 °C in 4% freshly prepared PFA, and 0 h PMD), yet all the antibodies tested allowed unequivocal identification of comparable densities of DCX^+^ immature neurons under these conditions (Supplementary Figs. 2–5). These cells presented an identifiable morphology, which was characterized by an oval-to-round relatively small soma located in the SGZ or the GCL, and a variable number of processes with distinct orientations^4,30^.

Prolonged PMD (F_2,40_ = 54.27; *p* < 0.001) and fixation (F_1,40_ = 286.4; *p* < 0.001) dramatically reduced the number of DCX^+^ immature neurons detected. Moreover, a statistically significant interaction between the two variables was observed (F_2,40_ = 54.27; *p* < 0.001), thereby revealing that the negative effects caused by prolonged PMD on the detection of these cells depend on fixation time and vice versa (Fig. 1b–h). Nevertheless, fixation time emerges as the most determinant factor impeding the detection of DCX^+^ immature DGCs, as none of these antibodies labeled intact numbers of positive cells in murine samples with 0 h PMD subjected to prolonged fixation (Supplementary Figs. 2–5), or in samples of human origin^29^. Interestingly, the application of an antigen retrieval protocol^29^ exclusively reversed the signal decay obtained with the two anti-DCX antibodies manufactured by Santa Cruz. The signal produced by the remaining antibodies did not show remarkable differences after antigen retrieval pre-treatment (Supplementary Fig. 7). In this regard, the epitope masking caused by prolonged fixation might differentially affect each of the epitopes against which these antibodies were raised. Conversely, distinct anti-DCX antibodies showed variable signal quality on murine samples subjected to artificial PMD intervals, their general performance being poorer as the PMD increased (Supplementary Figs. 2–5). Although the method of euthanasia did not influence the detection of DCX^+^ cells (Supplementary Fig. 8), the aforementioned results point to progressive degradation of DCX protein as the PMD increases. We confirmed the latter notion by performing WB analyses (Fig. 1i–j and Supplementary Fig. 9), which revealed the degradation of DCX protein (F_3,9_ = 192.2; *p* < 0.001) at 6 h (*p* = 0.021) and 24 h (*p* < 0.001) after death. Conversely, qPCR (Fig. 1k) showed that DCX messenger RNA (mRNA) was also degraded as a consequence of prolonged PMDs (F_2,9_ = 7.353; *p* = 0.013) but was preserved 6 h after death (*p* = 0.498). These data reveal the differential vulnerability of DCX protein and mRNA to post-mortem degradation.

We next examined another two well-characterized markers of immature DGCs, namely CR and PSA-NCAM. Both epitopes were sensitive to fixation duration (CR: F_1,42_ = 53.51; *p* < 0.001; PSA-NCAM: F_1,42_ = 15.49; *p* < 0.001) but not to the PMD (CR: F_2,42_ = 1.083; *p* = 0.348; PSA-NCAM: F_2,42_ = 0.289; *p* = 0.75) (Supplementary Fig. 1 and Supplementary data 1). Consistent with the higher sensitivity of DCX to both factors, the number of triple-labeled DCX^+^ CR^+^ PSA-NCAM^+^ immature DGCs decreased after prolonged fixation (F_1,42_ = 292.3; *p* < 0.001) and long artificial PMDs (F_2,42_ = 46.08; *p* < 0.001) (Fig. 1l). Similarly, the percentages of DCX^+^ cells that co-expressed either CR or PSA-NCAM decreased under these conditions (Supplementary Fig. 1 and Supplementary data 1).

Taken together, these data reveal that the detection of immature neurons requires short fixation times. Moreover, the visualization of DCX^+^ cells, in particular, is further negatively affected by prolonged PMD intervals in mice.

### Effects of fixation time and post-mortem delay (PMD) on the visualization of neural stem cells (NSC) and cells undergoing proliferation in the murine dentate gyrus (DG)

**Fig. 2:**
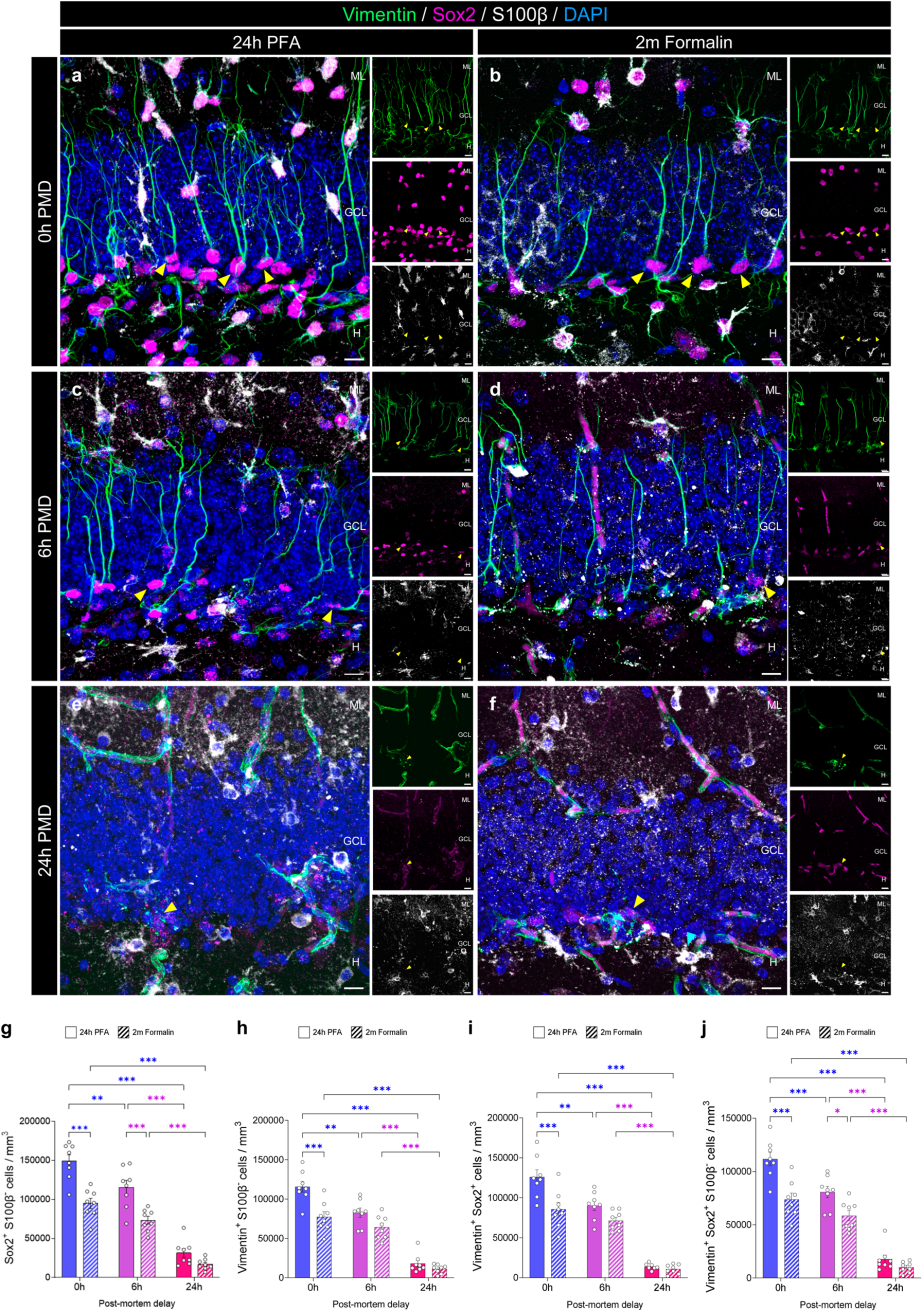
Effect of the post-mortem delay (PMD) and fixation time on the detection of neural stem cells (NSCs). **a**–**f** Representative images of SRY (sex determining region Y)-box 2 (Sox2), Vimentin, and S100 calcium-binding protein *β* (S100β) staining under distinct experimental conditions. **g** Density of Sox2^+^ S100β^-^ NSCs. **h** Density of Vimentin^+^ S100*β*^-^ NSCs. **i** Density of Vimentin^+^ Sox2^+^ cells. **j** Density of Vimentin^+^ Sox2^+^ S100β^-^ NSCs. In **a**–**f**, Z-projection images are shown. ML Molecular layer. GCL Granule cell layer. H Hilus. White scale bar: 10 μm. Yellow triangles: Vimentin^+^ Sox2^+^ S100*β*^-^ NSCs. Graphs represent mean values ± SEM. *n* = 8 mice. *0.05 > *p* ≥ 0.01; **0.01 > *p* ≥ 0.001; and ***p < 0.001.

We assessed whether prolonged fixation and PMD intervals interfered with the visualization of NSC markers such as Sox2 and Vimentin (Fig. 2a–f). To exclude the putative identification of astrocytes, only cells negative for the mature astrocyte marker S100β were considered^7^. Prolonged PMDs and fixation times led to a decrease in the number of Sox2^+^ S100β^-^ (Fixation: F_1,42_ = 47.56; *p* < 0.001; PMD: F_2,42_ = 118.3; *p* < 0.001), Vimentin^+^ S100β^-^ (Fixation: F_1,42_ = 22.86; *p* < 0.001; PMD: F_2,42_ = 119.6; *p* < 0.001), Vimentin^+^ Sox2^+^ (Fixation: F_1,41_ = 17.36; *p* < 0.001; PMD: F_2,41_ = 123; *p* < 0.001), and Vimentin^+^ Sox2^+^ S100β^-^ (Fixation: F_1,42_ = 30.88; *p* < 0.001; PMD: F_2,42_ = 131.9; *p* < 0.001) NSCs detected (Fig. 2g–j). Moreover, statistically significant interactions were found between PMD and fixation time (Supplementary data 1). This observation thus reveals that the effect of the PMD depends on the fixation time, as well as on the additive detrimental effects of these two factors on the visualization of NSCs in the murine hippocampus. The density of Ki67^+^ proliferative cells detected was affected by fixation time (F_1,42_ = 5.978; *p* = 0.019) but not by the PMD (F_2,42_ = 0.433; *p* = 0.651) (Supplementary Fig. 6a–m).

Therefore, fixation time also emerges as a critical factor for the visualization of NSCs and proliferative cells in the murine DG. Moreover, prolonged PMD intervals exert additive negative effects on the visualization of the former cells in this structure.

### Effects of prolonged post-mortem delay (PMD) intervals and fixation on the morphometric properties of the murine dentate gyrus (DG), and the numbers of mature dentate granule cells (DGCs) and glial cell subpopulations detected

**Fig. 3:**
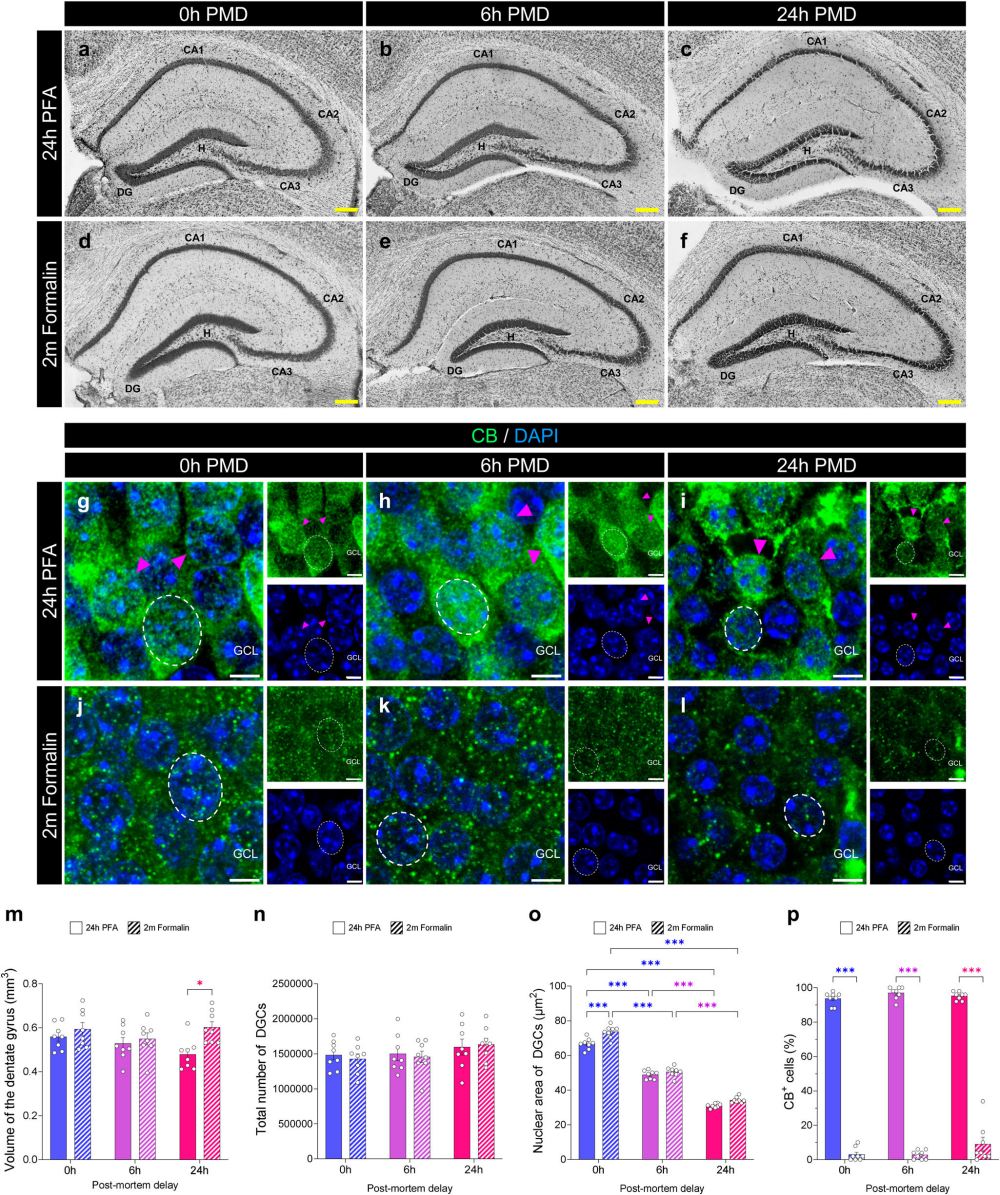
Effect of the post-mortem delay (PMD) and fixation time on the detection of markers of mature dentate granule cells (DGCs). **a**–**f** Representative images of Nissl staining showing the hippocampal anatomy under distinct experimental conditions. **g**–**l** High-power magnification images of Calbindin (CB) staining in the Granule cell layer (GCL) obtained under distinct experimental conditions. **m** Volume of the dentate gyrus (DG). **n** Total number of DGCs. **o** Nuclear area of DGCs. **p** Percentage of DGCs that express CB. In **g**–**l**, Z-projection images are shown. DG dentate gyrus. CA1-3: *Cornu Ammonis* 1 – 3. GCL Granule cell layer. H Hilus. Yellow scale bar: 200 μm. White scale bar: 5 μm. Magenta triangles: CB^+^ DGCs. Dotted line with circles: nuclear area. Graphs represent mean values ± SEM. *n* = 8 mice. *0.05 > p ≥ 0.01; and ***p < 0.001.

We analyzed the general anatomy of the DG by means of Nissl staining (Fig. 3a–f and m), which revealed no major effect of the PMD (F_2,42_ = 1.334; *p* = 0.274), but a significant influence of fixation time (F_1,42_ = 7.795; *p* = 0.008) on the volume of the DG (Fig. 3m). Neither the PMD (F_2,42_ = 1.905; *p* = 0.162) nor fixation time (F_1,42_ = 0.083; *p* = 0.774) modified the total number of DGCs counted (Fig. 3n). Conversely, both the PMD (F_2,42_ = 809.6; *p* < 0.001) and fixation time (F_1,42_ = 26.85; *p* < 0.001) affected the measured area of individual DGC nuclei (Fig. 3o). In this regard, nuclei of mice subjected to prolonged PMD appeared to be smaller and more dispersed within the GCL. The detected expression of CB in mature DGCs was almost abolished after a prolonged fixation (F_1,42_ = 3214; *p* < 0.001) but remained unaffected by the PMD (F_2,42_ = 1.878; *p* = 0.166) (Fig. 3g–l and p).

**Fig. 4:**
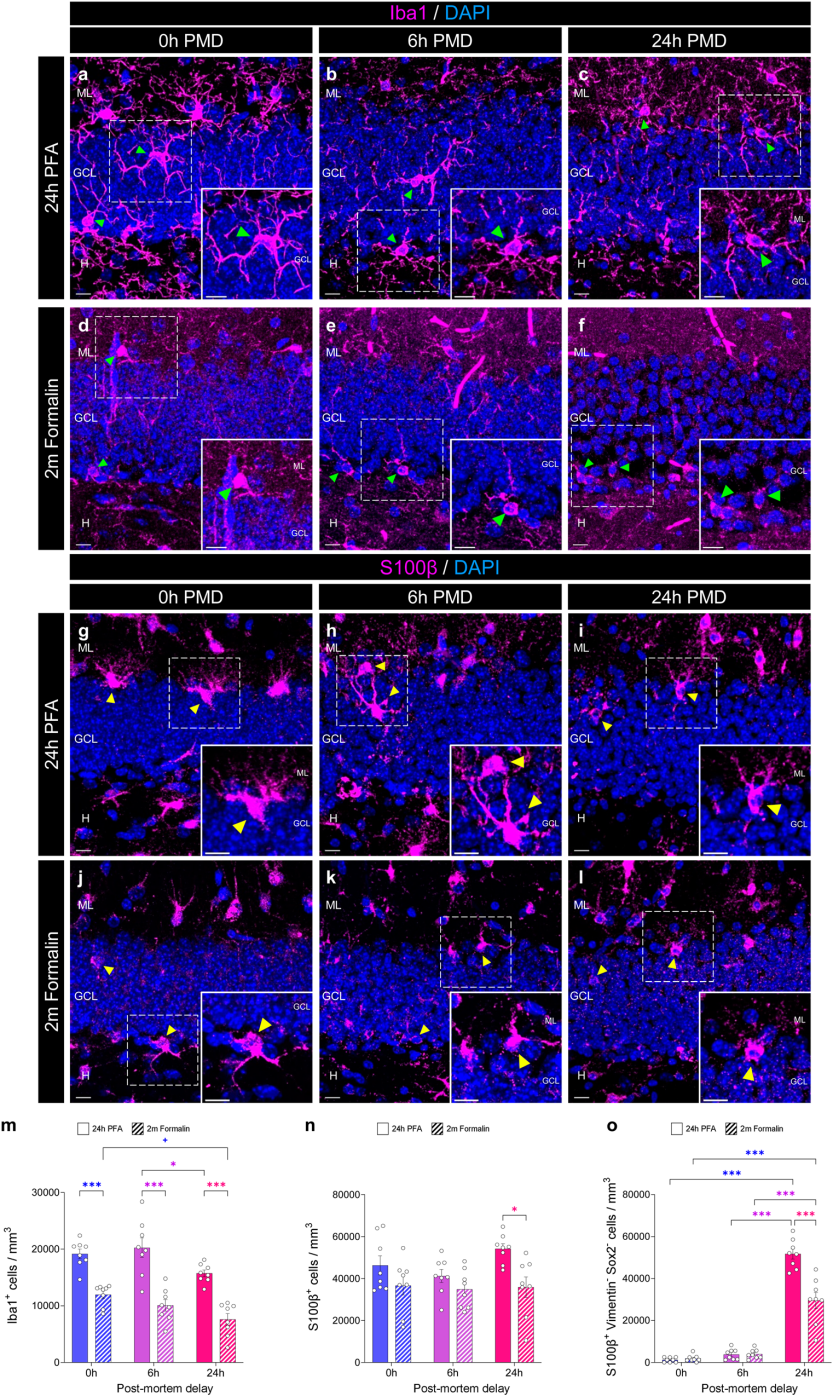
Effect of the post-mortem delay (PMD) and fixation time on the detection of glial cells. **a**–**f** Representative images of Iba1 staining showing microglial cells under distinct experimental conditions. **g**–**l** Representative images of S100 calcium-binding protein β (S100β) staining showing astrocytes under distinct experimental conditions. **m** Density of Iba1^+^ microglial cells. **n** Density of S100β ^+^ astrocytes. **o** Density of Sox2^-^ Vimentin^-^ S100*β* ^+^ mature astrocytes. In **a**–**l**, Z-projection images are shown. ML Molecular layer. GCL Granule cell layer. H Hilus. Sox2 SRY (sex determining region Y)-box 2. White scale bar: 10 μm. Green triangles: Iba1^+^ microglial cells. Yellow triangles: S100*β*^+^ astrocytes. Graphs represent mean values ± SEM. *n* = 8 mice. +0.1 > *p* ≥ 0.05; *0.05 > *p* ≥ 0.01; and ***p < 0.001.

The density of the Iba1^+^ microglial cells (Fig. 4a–f and m) counted was reduced after prolonged PMDs (F_2,42_ = 8.247; *p* < 0.001) and fixation times (F_1,42_ = 97.100; *p* < 0.001). The number of S100β^+^ astrocytes was modified by the fixation time (F_1,42_ = 12.910; *p* < 0.001) but not by the PMD (F_2,42_ = 1.607; *p* = 0.213) (Fig. 4g–l and n). Given the aforementioned sensitivity of Vimentin and Sox2 to both the PMD and fixation time, the number of S100β^+^ Vimentin^-^ Sox2^-^ fully mature astrocytes was affected by both parameters (Fixation: F_1,42_ = 20.080; *p* < 0.001; PMD: F_2,42_ = 252.900; *p* < 0.001) (Fig. 4o). Moreover, not only prolonged PMD but also long fixation caused a dramatic decrease in Iba1 and S100β signal quality since no labeling of distal processes was observed in samples subjected to prolonged fixation and/or artificial PMD intervals.

Taken together, these results indicate that prolonged fixation impedes the visualization of markers of immature and mature DGCs, NSCs, proliferative cells, microglia, and astrocytes, as well as altering the results of distinct morphometric determinations in mice. Moreover, prolonged PMDs further complicate the visualization of particularly sensitive epitopes such as DCX, Sox2, Vimentin, and Iba1 (Table 1). The PMD and fixation time exert additive detrimental effects on the signal quality of all the epitopes analyzed.

**Table 1:** Main effects of the post-mortem delay (PMD) and fixation time on the detection of markers specific to distinct stages of adult hippocampal neurogenesis (AHN).

Markers		Cell density			Quality of the signal	
		Effect of PMD	Effect of Fixation	Interaction	Effect of PMD	Effect of Fixation
Immature Neurons	DCX (#326004)	Decreased	Abolished	Yes	Decreased	Abolished
	CR (#CR-7697)	Maintained	Decreased	No	Decreased	Decreased
	PSA-NCAM (#mab5324)	Maintained	Decreased	Yes	Maintained	Decreased
Neural Stem Cells	Vimentin (#ab193555)	Decreased	Decreased	Yes	Decreased	Decreased
	Sox2 (#AF2018)	Decreased	Decreased	Yes	Decreased	Decreased
Mature DGCs	CB (#C9848)	Maintained	Abolished	No	Maintained	Abolished
Astrocytes	S100β (#287004)	Maintained	Decreased	No	Decreased	Decreased
Microglia	Iba1 (#234006)	Decreased	Decreased	No	Decreased	Decreased
Proliferative cells	Ki67 (#ab15580)	Maintained	Decreased	No	Decreased	Decreased

The effects observed in two-way ANOVA statistical comparisons are shown. *DCX* Doublecortin, *CR* Calretinin, *PSA-NCAM* Polysialylated-neural cell adhesion molecule. *Sox2 SRY* (sex determining region Y)-box 2. *S100β* S100 calcium-binding protein β, *DGCs* dentate granule cells.

## Discussion

AHN takes place in more than 120 mammalian species (reviewed in ref. ^18^), although the occurrence of this phenomenon was questioned in northern minke whales and harbor porpoises^34^, and in a small number of echolocating microbats captured from the wild^21^. However, subsequent determinations attributed the putative absence of AHN marker detection in the latter species to technical, environmental, and ante- and peri-mortem factors^19,20^, thereby preventing further replication of these negative results. Since 1998^24^, more than 40 studies (summarized in Table 2 of ref. ^18^) that applied distinct methodologies (IHC, C^14^ incorporation, functional magnetic spectroscopy, and single-cell RNAseq, among others^25–27,30,35,36^) have confirmed the occurrence of AHN in humans. However, a study published in 2018 questioned the presence of AHN markers in the human DG^28^, thereby raising a so-called *controversy* regarding the demonstration of AHN in our species^37–39^. In this regard, using post-mortem human hippocampal samples collected under tightly controlled conditions, we assessed the impact of tissue processing methodologies on the detection of AHN markers in the human DG^29,30,40^. Our studies revealed that prolonged fixation impedes the visualization of immature neurons in this structure and, therefore, that human post-mortem hippocampal samples subjected to long aldehyde fixation are not suitable for the study of AHN^30^. In fact, none of the nine antibodies tested detected DCX^+^ immature DGCs in human hippocampal samples fixed for six months in formalin, whereas they provided high-quality signal in samples obtained from the same subjects fixed for 24 h^29^. Similarly, prolonged fixation of mouse brain samples that lack a PMD significantly decreased the quality of the signal obtained with 10 distinct anti-DCX antibodies (Supplementary Figs. 2–5), thereby impeding the detection of cell densities comparable to those obtained upon 24 h fixation. In fact, prolonged fixation prevented the immunohistochemical detection of DCX protein by several of the anti-DCX antibodies tested. Antigen retrieval protocols showed very limited effectiveness in reversing signal decay for most of the antibodies tested (Supplementary Fig. 7). Therefore, in light of these results, fixation emerges as a methodological cornerstone that can affect researchers´ capacity to visualize immature neurons in the mammalian DG.

In a previous human AHN study^30^, we did not observe a statistically significant correlation between the density of DCX^+^ immature neurons counted and the PMD. These negative results may be related to the fact that we used human hippocampal samples with relatively short PMDs (10.42 h ± 2.809 h; Minimum: 3 h; Maximum: 38 h). Indeed, only two samples had PMDs longer than 20 h. In this regard, some studies showed negative effects caused by the PMD on DCX detection in rats^31,32^, which led several authors to deem the use of human samples with prolonged PMD intervals problematic^37,38^. The data presented here strongly support the negative effect of prolonged PMDs on the visualization of murine DCX^+^ immature DGCs (Fig. 1 and Supplementary Figs. 1–5). In this respect, prolonged PMD not only sharply reduced the number of DCX^+^ immature neurons detected but also markedly diminished signal quality and distal dendritic staining. Six hours after the death of the animals, these effects were evident for eight out of the ten anti-DCX antibodies tested, and at 24 h after death, they were noticeable for all of them. However, the existence of a statistically significant interaction with the fixation time reveals that the effects of the PMD on the detection of murine DCX^+^ immature neurons do indeed depend on the length of the fixation process.

Distinct factors might underlie the apparent dissimilar influence of the PMD on the detection of DCX^+^ immature neurons in mice and humans. First, putative inter-species differences in antibody sensitivity^41^, protein degradation speed, preservation of specific epitope domains, among others, cannot be ruled out. Second, differences in body size may result in changes in brain cooling rates after death, which, in turn, might affect the activity of degrading enzymes. Additionally, given that aldehyde fixatives penetrate the tissue at a rate of ~1 mm per h^42^, differences in the size of murine and human brain samples might also influence protein degradation before the fixative achieves full penetration in the whole tissue block. Third, in our previous study in humans^30^, we determined the influence of the PMD on the detection of DCX^+^ immature neurons in samples fixed for 24 h, in which overall antigenicity was preserved. The statistically significant interaction between the PMD and fixation time described here suggests that the short fixation of those samples might contribute to attenuating the negative effects of a prolonged PMD. Fourth, the detection of AHN markers in human samples fixed for 24–48 h requires the application of a mild pre-treatment protocol (based on the pre-incubation of the tissue with sodium borohydride (NaBH_4_) and a heat-mediated citrate buffer antigen retrieval before IHC) to reduce autofluorescence^29^. Although these pre-treatments are not necessary for human samples fixed for shorter periods or mouse tissue^30^, their application may render partially degraded epitopes that are more accessible to antibodies, thereby putatively mitigating some of the negative consequences of prolonged PMDs in our previous study^30^. Fifth, the data presented here reveal that distinct anti-DCX antibodies show not only variations in signal quality under *ideal* tissue processing conditions (namely 24 h of PFA fixation and 0 h PMD) but also marked differential susceptibility to prolonged PMD intervals. The observation that one of the most widely used antibodies in AHN studies, namely the discontinued goat anti-DCX antibody from Santa Cruz (Catalog number #8066), did not appear to be substantially affected by the PMD in human hippocampal tissue^30^ does not exclude the possibility that other anti-DCX antibodies exhibit a more pronounced decay in signal specificity and intensity in the same species, as occurs in mice (Supplementary Figs. 2–5). This aspect gains special relevance in the context of human studies, in which an exhaustive validation of each antibody used is crucial^43^. Methods allowing the selective labeling of the whole morphology of human immature DGCs, which are not yet available, might detect previously unnoticed putative reductions in distal dendritic staining of DCX^+^ cells and/or the impoverishment of signal quality caused by prolonged PMD in human tissue.

DCX protein can be partially detected by IHC 6 h after death—an observation that is in agreement with its incipient degradation observed by WB (Fig. 1i–j). Similarly, the immunostaining profiles detected for numerous proteins did not appear to be affected by ≥50 h PMD, despite the observation of degradation patterns in WB^44^. Remarkably, unlike other mRNA transcripts^45^, that of DCX showed a trend towards enhanced vulnerability to 24 h of post-mortem degradation (Fig. 1k). In this regard, several pre-mortem factors, such as age and sex, or brain pH, among others, influence the amount and quality of brain RNA in human subjects^46^. Moreover, the length of the agonal state selectively influences the degradation of some messenger RNAs in humans^47^. These results further challenge the conclusions of a recent study using scRNAseq that questioned the occurrence of human AHN^48^. In fact, other published work that used shorter PMDs succeeded in fully reconstructing human AHN trajectories using equivalent methodologies^36,49^. A recent study that re-analyzed various published human scRNAseq datasets highlighted the importance of methodological, conceptual, and biological factors, which can bias transcriptomic data analysis and interpretation^50^.

Our results bring to light the particularly sensitive nature of DCX protein to both fixation and post-mortem degradation. However, whether these factors affect the detection of other AHN markers in a similar manner has not been addressed to date. Our findings reveal that some of the markers most widely used to identify individual AHN stages (including markers of immature neurons (such as CR, and PSA-NCAM), NSCs (like Sox2 and Vimentin), proliferative cells (Ki67), and mature DGCs (CB)) are highly vulnerable to prolonged fixation and that certain epitopes (such as Sox2 and Vimentin) are, in addition, negatively affected by dilated PMD intervals in mice (Table 1). The susceptibility of these markers to prolonged fixation is supported by human studies^30,40^, although no correlation between the number of cells detected and the PMD was observed in that species (Supplementary Fig. S14 in ref. ^40^ and Extended data Fig. 1 in ref. ^30^). In this regard, similar (and/or alternative) causes to those suggested to underlie the differential effect of the PMD on the detection of DCX protein in mice and humans may also play a role. For instance, in contrast to mice, the visualization of NSC markers in the adult human DG requires the use of mild detergents^40^, which might reflect the differentially labile nature of these epitopes in these two species. In addition, previous data show remarkable inter-species differences in immature DGC marker expression^51^, which should be taken into account in comparative studies. Moreover, in the light of previous data published on rats^32^, a differential interaction between age and the PMD in distinct species might take place.

In summary, the results presented here bring to light that the use of prolonged (or uncontrolled) fixation protocols significantly decreases the reliability of AHN studies. An inadequate fixation protocol might not only impede the visualization of cells positive for distinct AHN markers, but also dramatically diminish signal specificity and quality. Moreover, particularly labile epitopes might also show enhanced vulnerability to post-mortem degradation (Table 1). This study emphasizes the importance of controlling key methodological aspects related to the use of post-mortem brain samples. Our results gain further relevance in the context of AHN studies in humans, non-human primates, and wild-living organisms, in which neither the PMD nor the length of the fixation protocol is fully controllable, known, and/or reported in detail. Overlooking such technical aspects may lead to biased (or incorrect) conclusions that merely rely on a the artefactual absence of evidence for a given phenomenon.

## Methods

### Experimental design

To study whether prolonged fixation and long PMD exert either independent or additive negative effects on the visualization of markers of distinct stages of AHN, we used a tightly controlled experimental design in mice (Fig. 1a). We included three groups of seven- to nine-week-old female mice (12 in each group). Two of these groups were sacrificed by carbon dioxide inhalation and subjected to artificially generated PMD intervals of 6 h and 24 h respectively, whereas a control group was anesthetized and intracardially perfused with saline and therefore lacked a PMD. Eight mice from each experimental group were used for histological analysis, including immunohistochemistry (IHC) and Nissl staining. In this regard, the right hemisphere of the brains of these animals was fixed in freshly prepared 4% paraformaldehyde (PFA) for 24 h at 4 °C, whereas the left hemisphere was fixed in a commercial 3.7% formaldehyde solution for 2 months at room temperature (rt). This approach allowed the intra-individual assessment of the independent and/or additive effects of the PMD and prolonged fixation on the study of AHN. The remaining four mice from each experimental group were used for Western blot (WB) (right hemisphere) and quantitative polymerase chain reaction (qPCR) (left hemisphere) determinations. To determine whether the method of euthanasia influences the detection of markers of AHN, we included a fourth group of mice, which were sacrificed by carbon dioxide inhalation but not subjected to a PMD. These animals were compared with those anesthetized and intracardially perfused with saline.

### Animals

Five- to seven-week-old female C57BL/6J-OlaHsd mice were obtained from Envigo Laboratories. They were housed in the animal facility at the *Centro de Biología Molecular Severo Ochoa* (CBMSO) following European Community Guidelines (directive 86/609/EEC) and handled following European and local animal care protocols. The mice were subjected to at least one week of habituation before the experiments began. Four mice were housed per cage. Given that hierarchy/ dominance relationships between male mice have a negative impact on AHN^52^, only female mice were used in this study. As the fluctuations in the endogenous levels of estradiol occurring during the estrous cycle have been reported not to influence the levels of AHN in adult female C57BL/6 mice^53^, the estrous cycle was not synchronized in these animals. All animal experiments were approved by the CBMSO (AEEC-CBMSO-23/172) and National (PROEX 185.4/20) Ethics Committees. We have complied with all relevant ethical regulations for animal testing.

### Sacrifice

The animals belonging to the 0 h PMD group were fully anesthetized by an intraperitoneal injection of pentobarbital (EutaLender, 60 mg/kg) and transcardially perfused with 0.9% saline. The mice subjected to artificially generated PMD were sacrificed by carbon dioxide inhalation and kept at rt for either 6 h or 24 h. An additional group of mice was sacrificed by carbon dioxide inhalation but not subjected to a PMD. The brains of the mice used for IHC were removed from the skulls and the hemispheres were separated. The two hemispheres were fixed separately in commercial fixative solutions that contained a 4% final formaldehyde concentration. Following a previously described protocol specifically optimized for the visualization of AHN markers^29,30^, the right hemisphere was fixed in 4% PFA (pH  =  7.4) diluted in 0.1 N phosphate buffer (PB) for 24 h at 4 °C. This fixative solution was prepared from a commercial 16% PFA solution (Electron Microscopy Sciences, #15710). Following the standard procedure used by most brain banks worldwide, the left hemisphere was fixed in a commercial 4% formaldehyde solution (pH = 7.1) (Sigma-Aldrich, #HT501128-4L) for 2 months at rt. After fixation, hemispheres were washed three times in 0.1 N PB. The brains of mice used for WB and qPCR were removed from the skulls and the hippocampi were rapidly dissected on ice, frozen in dry ice, and kept at −80 °C.

### Brain tissue sectioning

For IHC determinations, brain hemispheres were included in a 10% sucrose-4% agarose solution^29,30,40^, and 50 µm-thick coronal sections were obtained on a Leica VT1200S vibratome (Leica Biosystems, Wetzlar, Germany). Series of brain slices were randomly made up of one section from every ninth. For each series of sections, the sampling probability was 1/8. Brain sections were immediately stored at −20 °C in 24-well plastic plates filled with a cryopreservative solution (30% polyethylene glycol; 10% 0.2 N PB; 30% glycerol; and 30% bi-distilled water).

### Sodium borohydride (NaBH_4_) and heat-mediated citrate buffer antigen retrieval (HC-AR) incubation procedures

Sections were immersed in 1 ml of 0.5% NaBH_4_ solution diluted in PB 0.1 M for 30 min at rt and under gentle shaking. HC-AR was performed in 10-ml glass scintillation vials. Sections belonging to the same animal were immersed in 5 ml of a preheated 1 × citrate buffer (pH 6.0) antigen retrieval solution (a commercial 10 × stock solution (Vector, H-3300) was diluted in distilled water). The vials were exposed to 5–6 brief (10–20 s) cycles of microwave heating. Boiling of the liquid was avoided to prevent tissue damage. Next, the vials were immersed in an 80 °C water bath for 20 min and subsequently left at rt for 20 min. Afterwards, sections were rinsed five times in 0.1 N PB, and immunohistochemistry was performed as described below.

### Immunohistochemistry (IHC)

Briefly, sections were rinsed in 0.1 N PB at rt. Triple IHC was performed as described previously^54^. The incubation buffer for all primary (Supplementary Table 1) and secondary (Supplementary Table 2) antibodies contained 1% Triton X-100 and 1% bovine serum albumin (BSA) diluted in 0.1 N PB. Incubation with the primary antibodies was performed under gentle shaking at 4 °C for between 48 h and 72 h. To detect the binding of primary antibodies, Alexa®-coupled fluorescent secondary antibodies were used at a concentration of 1:1000 and incubated for 24 h at 4 °C. After incubation, the sections were rinsed three times in 0.1 N PB and stained for 10 min with 4′,6-diamidino-2-phenylindole (DAPI) (Merck, 1:5000) to label nuclei. They were then mounted on gelatine-coated glass slides. A non-commercial anti-fading mounting medium (33% glycerol and 7.5% Mowiol, prepared in Tris-HCl 0.2 M pH = 8.5) was used for embedding the sections.

### Western blot (WB)

Extracts for WB analysis were prepared by homogenizing the hippocampus in an ice-cold extraction buffer consisting of 50 mM Tris HCl, pH 7.4, 150 mM NaCl, 1% Triton, 0.5% Deoxycholate, 0.1% SDS, 1 mM NaF, 1 mM sodium orthovanadate, 1 µM okadaic acid and a protease inhibitor cocktail (Roche, #11697498001). Next, protein content was determined by the Pierce BCA Protein Assay (Thermo Fisher, #23225) method and 25 μg of total protein was electrophoresed on 10% SDS-polyacrylamide gel and transferred to a nitrocellulose membrane (Amersham Biosciences, #10600002). The membranes were incubated in blocking solution containing 5% non-fat dried milk diluted in PBS-Tween 0.1% at rt for 1 h. Primary antibodies (Supplementary Table 1) were incubated at 4 °C overnight in the same solution. A secondary anti-rabbit antibody (Supplementary Table 2) was incubated for 2 h at rt, and enhanced chemiluminescence (ECL) detection reagents (Perkin Elmer, #NEL105001EA) were used for immunodetection. The chemiluminescent signal was captured in an ImageQuant™ LAS 4000 mini (GE Healthcare, Illinois, USA) and quantified using *Fiji* software. Values were first normalized with respect to the expression of Vinculin to correct for total protein content. The data were subsequently normalized with respect to the 0 h PMD control group.

### RNA extraction and cDNA synthesis

RNA was isolated from each hippocampal sample using TRIzol^®^ (Invitrogen, #15596026) following the instructions provided by the manufacturer. The RNA pellet was resuspended in 30 µl of RNase-free water. RNA concentration and purity were determined using a NanoDrop One spectrophotometer (Thermo Fisher Scientific, Massachusetts, USA). After RNA isolation, cDNA synthesis was carried out using the RevertAid H Minus First Strand cDNA Synthesis Kit (Thermo Fisher Scientific, #K1632), and 500 ng of RNA from each sample was combined with the Master Mix containing 5X Reaction Buffer, RiboLock RNase Inhibitor (20 U/µl), 10 mM dNTP Mix, Random Primers, and RevertAid HMinus M-MuLV Reverse Transcriptase (200 U/µl) to a final volume of 20 µl. Samples were then immediately incubated in a thermocycler for 5 min at 25 °C, 60 min at 42 °C, and 5 min at 70 °C. Synthesized cDNA was kept at −20 °C and −80 °C for short- and long-term storage, respectively.

### Quantitative polymerase chain reaction (qPCR)

The GoTaq^®^ qPCR Master Mix (Promega, #A6001) was used to carry out qPCR. Briefly, 0.2 µl of synthesized cDNA was combined with the Master Mix, forward and reverse primers, carboxy-X-rhodamine (CXR) reference dye, and nuclease-free water to a final volume of 10 µl and loaded on 384-white-well PCR plates (Bio-Rad, #HSP3805). Data were collected on a CXF-384 real-time PCR detection system (Bio-Rad Laboratories, California, USA). Samples were incubated for 2 min at 95 °C followed by 40 cycles of 15 sec at 95 °C and 1 min at 60 °C. All measurements were made in triplicate, and controls for non-template and genomic DNA were included. The following intron-spanning primers were used to detect DCX (ref. ^55^: forward 5’-CAGTCAGCTCTCAACACCTAAG-3’ and reverse 5’-CATCTTTCACATGGAATCGCC-3’), and housekeeping Glyceraldehyde 3-phosphate dehydrogenase ((GAPDH)^56^: forward 5’-AGGTCGGTGTGAACGGATTTG-3’ and reverse 5’-GGGGTCGTTGATGGCAACA-3’). Primer efficiency was previously assessed by a six-point standard curve using serially diluted pooled cDNA. GAPDH gene was used as a reference, and fold change was calculated with respect to the 0 h PMD control group of mice using the ΔΔCT method.

### Nissl staining

A randomly chosen series was used to calculate the total volume of the DG in each animal using Nissl staining. Slices were mounted on 2% gelatine-coated glass slides and air-dried at rt for 48 h. The slides were sequentially immersed in the following solutions: 6 min in toluidine blue, 10 sec in bi-distilled water, 2 min in EtOH 70°, 2 min in EtOH 96°, 2 min in EtOH 100°, 2 min in EtOH 100°, and 2 min in Xylene (PanReac, #251769). Next, sections were embedded in DePex (Serva Electrophoresis™, #1824301) and dried for 48 h at rt before imaging. Images were acquired under a THUNDER Imager Tissue microscope equipped with a Leica DFC9000 GTC VSC-09991 camera (Leica Microsystems Ltd., Wetzlar, Germany) and using a 5X dry objective. Images were processed using the Leica Application Suite X (LAS X) software provided by the manufacturer (Leica Microsystems Ltd., Wetzlar, Germany). The DG volume was determined using the freehand selection tool in *Fiji* software to measure the granule cell layer (GCL) plus the SGZ area on each section of the series. Each area was multiplied by the thickness of the tissue (namely 50 µm) and by the sampling fraction (8). The numbers obtained were summed to calculate the total DG volume, which is expressed in mm^3^.

### Cell counts

To determine the density of CR^+^, DCX^+^, PSA-NCAM^+^, Vimentin^+^, Sox2^+^, S100β^+^, Iba1^+^, and Ki67^+^ cells, six stacks of images containing the DG were obtained using an LSM900 Zeiss confocal microscope (Zeiss, Oberkochen, Germany) (for Vimentin^+^, Sox2^+^, S100β^+^, Iba1^+^, and Ki67^+^: 40X oil immersion objective; XY dimensions: 159.72 µm; Z-interval: 1.4 µm, and for CR^+^, DCX^+^, PSA-NCAM^+^: 63X oil immersion objective; XY dimensions: 101.41 µm; Z-interval: 1.4 µm). The density of positive cells was estimated using unbiased stereology methods based on the use of the physical dissector method adapted to confocal microscopy^29,30,40,57^. Briefly, for Vimentin^+^, Sox2^+^, S100β^+^, Iba1^+^, and Ki67^+^ cells, an area containing the region of interest (namely the GCL plus the SGZ) was traced on the DAPI channel of each confocal stack of images using the freehand drawing tool of *Fiji*, and the area of each of these structures was then calculated. Areas were multiplied by the z-thickness of the stack to calculate the reference volume^29,30^. For CR^+^, DCX^+^, PSA-NCAM^+^ stacks, the length of the SGZ was measured on the DAPI channel of each confocal stack of images using the freehand line drawing tool of *Fiji*. The lengths were then multiplied by the z-thickness of the stack to calculate the reference area, namely the SGZ^57^. Next, to calculate the density of positive cells (number of cells/mm^3^ or cells/mm^2^), the number of cells positive for each marker was counted on individual planes and then divided by the reference volume/area of the stack. To determine the density, CB expression, and nuclear area of DGCs, six stacks of images containing the DG were obtained using an LSM900 Zeiss confocal microscope (63X oil immersion objective; zoom: 3; XY dimensions: 33.8 µm; Z-interval: 1.4 µm). DGCs were counted on individual planes and the number of cells was divided by the reference volume to calculate the density (number of cells/mm^3^). The total number of DGCs was then calculated for each animal by multiplying the DGC density by the DG volume. The percentage of DGCs that expressed CB was determined on 50 cells per animal, randomly selected on the DAPI channel, with DGC nuclear morphology. The percentage of CB^+^ DGCs was then calculated for each animal. The nuclear area of 50 DGCs per mouse was measured in the central plane using the freehand selection tool of *Fiji*.

### Statistics and reproducibility

Statistical analyses were performed using the GraphPad Prism 9 software (GraphPad.v.9.4.1 (681), 2022; GraphPad Software, LLC). The normality of sample distribution was assessed using a Kolmogorov–Smirnov test and extreme outlier values were eliminated when necessary. For data in which one variable was being analyzed, a one-way ANOVA followed by a Tukey post-hoc test was used. For comparisons in which two variables were analyzed, a two-way ANOVA test was used. A Tukey post-hoc test was used to compare individual groups. Graphs represent mean values ± SEM. A 95% confidence interval was used for statistical comparisons. The detailed results of statistical comparisons are included in the Supplementary Data 1 file.

### Reporting summary

Further information on research design is available in the Nature Portfolio Reporting Summary linked to this article.

### Supplementary information

Supplementary material

Description of Additional Supplementary Files

Supplementary Data 1 and 2

Reporting Summary

## Data Availability

All the data generated in this study have been included in the Main or Supplementary Figures and are available from the corresponding author upon reasonable request. Source data is included in the Supplementary Data 2 file. Unprocessed gel images are shown in Supplementary Fig. 9.

## References

[1] Altman J (1963). Autoradiographic investigation of cell proliferation in the brains of rats and cats. Anat. Rec..

[2] Toni N (2007). Synapse formation on neurons born in the adult hippocampus. Nat. Neurosci..

[3] Llorens-Martin M, Jurado-Arjona J, Avila J, Hernandez F (2015). Novel connection between newborn granule neurons and the hippocampal CA2 field. Exp. Neurol..

[4] Kempermann G, Jessberger S, Steiner B, Kronenberg G (2004). Milestones of neuronal development in the adult hippocampus. Trends Neurosci..

[5] Seri B, Garcia-Verdugo JM, McEwen BS, Alvarez-Buylla A (2001). Astrocytes give rise to new neurons in the adult mammalian hippocampus. J. Neurosci..

[6] Moss J (2016). Fine processes of Nestin-GFP-positive radial glia-like stem cells in the adult dentate gyrus ensheathe local synapses and vasculature. Proc. Natl. Acad. Sci. USA.

[7] Martin-Suarez S, Valero J, Muro-Garcia T, Encinas JM (2019). Phenotypical and functional heterogeneity of neural stem cells in the aged hippocampus. Aging Cell.

[8] Harris L (2021). Coordinated changes in cellular behavior ensure the lifelong maintenance of the hippocampal stem cell population. Cell Stem Cell.

[9] Bottes S (2021). Long-term self-renewing stem cells in the adult mouse hippocampus identified by intravital imaging. Nat. Neurosci.

[10] Plumpe, T. et al. Variability of doublecortin-associated dendrite maturation in adult hippocampal neurogenesis is independent of the regulation of precursor cell proliferation. *BMC Neurosci.*10.1186/1471-2202-7-77 (2006). 10.1186/1471-2202-7-77 PMC1657022 17105671

[11] des Portes V (1998). A novel CNS gene required for neuronal migration and involved in X-linked subcortical laminar heterotopia and lissencephaly syndrome. Cell.

[12] Gleeson JG, Lin PT, Flanagan LA, Walsh CA (1999). Doublecortin is a microtubule-associated protein and is expressed widely by migrating neurons. Neuron.

[13] Francis F (1999). Doublecortin is a developmentally regulated, microtubule-associated protein expressed in migrating and differentiating neurons. Neuron.

[14] Brandt MD (2003). Transient calretinin expression defines early postmitotic step of neuronal differentiation in adult hippocampal neurogenesis of mice. Mol. Cell. Neurosci..

[15] Zhao C, Teng EM, Summers RG, Ming GL, Gage FH (2006). Distinct morphological stages of dentate granule neuron maturation in the adult mouse hippocampus. J. Neurosci..

[16] Chancey JH, Poulsen DJ, Wadiche JI, Overstreet-Wadiche L (2014). Hilar mossy cells provide the first glutamatergic synapses to adult-born dentate granule cells. J. Neurosci..

[17] Overstreet Wadiche L, Bromberg DA, Bensen AL, Westbrook GL (2005). GABAergic signaling to newborn neurons in dentate gyrus. J. Neurophysio..

[18] Terreros-Roncal, J. et al. Methods to study adult hippocampal neurogenesis in humans and across the phylogeny. *Hippocampus*10.1002/hipo.23474 (2022). 10.1002/hipo.23474 PMC7614361 36259116

[19] Chawana R (2020). Adult hippocampal neurogenesis in egyptian fruit bats from three different environments: Are interpretational variations due to the environment or methodology?. J. Comp. Neurol..

[20] Chawana R (2014). Microbats appear to have adult hippocampal neurogenesis, but post-capture stress causes a rapid decline in the number of neurons expressing doublecortin. Neurosci..

[21] Amrein I, Dechmann DK, Winter Y, Lipp HP (2007). Absent or low rate of adult neurogenesis in the hippocampus of bats (Chiroptera). PLoS One.

[22] Sorrells SF (2021). Positive controls in adults and children support what very few, If any, new neurons are born in the adult human hippocampus. J. Neurosci..

[23] Moreno-Jimenez EP, Terreros-Roncal J, Flor-Garcia M, Rabano A, Llorens-Martin M (2021). Evidences for adult hippocampal neurogenesis in humans. J. Neurosci..

[24] Eriksson PS (1998). Neurogenesis in the adult human hippocampus. Nat. Med..

[25] Spalding KL (2013). Dynamics of hippocampal neurogenesis in adult humans. Cell.

[26] Knoth R (2010). Murine features of neurogenesis in the human hippocampus across the lifespan from 0 to 100 years. PLoS One.

[27] Boldrini M (2018). Human hippocampal neurogenesis persists throughout aging. Cell Stem Cell.

[28] Sorrells SF (2018). Human hippocampal neurogenesis drops sharply in children to undetectable levels in adults. Nature.

[29] Flor-Garcia M (2020). Unraveling human adult hippocampal neurogenesis. Nat. Protoc..

[30] Moreno-Jimenez EP (2019). Adult hippocampal neurogenesis is abundant in neurologically healthy subjects and drops sharply in patients with Alzheimer’s disease. Nat. Med..

[31] Boekhoorn K, Joels M, Lucassen PJ (2006). Increased proliferation reflects glial and vascular-associated changes, but not neurogenesis in the presenile alzheimer hippocampus. Neurobiol. Dis..

[32] Terstege DJ, Addo-Osafo K, Campbell Teskey G, Epp JR (2022). New neurons in old brains: implications of age in the analysis of neurogenesis in post-mortem tissue. Mol. Brain.

[33] Klioueva N, Bovenberg J, Huitinga I (2017). Banking brain tissue for research. Handb. Clin. Neurol.

[34] Patzke N (2015). In contrast to many other mammals, cetaceans have relatively small hippocampi that appear to lack adult neurogenesis. Brain Struct. Funct..

[35] Manganas LN (2007). Magnetic resonance spectroscopy identifies neural progenitor cells in the live human brain. Science.

[36] Zhou, Y. et al. Molecular landscapes of human hippocampal immature neurons across lifespan. *Nature*10.1038/s41586-022-04912-w (2022). 10.1038/s41586-022-04912-w PMC9316413 35794479

[37] Kempermann G (2018). Human adult neurogenesis: evidence and remaining questions. Cell Stem Cell.

[38] Lucassen PJ (2020). Limits to human neurogenesis-really?. Mol. Psychiatry.

[39] Lucassen PJ, Fitzsimons CP, Salta E, Maletic-Savatic M (2020). Adult neurogenesis, human after all (again): classic, optimized, and future approaches. Behav. Brain Res..

[40] Terreros-Roncal J (2021). Impact of neurodegenerative diseases on human adult hippocampal neurogenesis. Science.

[41] Ghibaudi, M. et al. Consistency and variation in doublecortin and Ki67 antigen detection in the brain tissue of different mammals, including humans. *Int. J. Mol. Sci.*10.3390/ijms24032514 (2023). 10.3390/ijms24032514 PMC9916846 36768845

[42] Thavarajah R, Mudimbaimannar VK, Elizabeth J, Rao UK, Ranganathan K (2012). Chemical and physical basics of routine formaldehyde fixation. J. Oral Maxillofac Pathol..

[43] Liu RX (2020). No DCX-positive neurogenesis in the cerebral cortex of the adult primate. Neural Regen. Res..

[44] Blair JA (2016). Individual case analysis of postmortem interval time on brain tissue preservation. PLoS One.

[45] Perrett CW, Marchbanks RM, Whatley SA (1988). Characterisation of messenger RNA extracted post-mortem from the brains of schizophrenic, depressed and control subjects. J. Neurol. Neurosurg. Psychiatry.

[46] Preece P, Cairns NJ (2003). Quantifying mRNA in postmortem human brain: influence of gender, age at death, postmortem interval, brain pH, agonal state and inter-lobe mRNA variance. Brain Res. Mol. Brain Res..

[47] Barton AJ, Pearson RC, Najlerahim A, Harrison PJ (1993). Pre- and postmortem influences on brain RNA. J. Neurochem..

[48] Franjic D (2022). Transcriptomic taxonomy and neurogenic trajectories of adult human, macaque, and pig hippocampal and entorhinal cells. Neuron.

[49] Wang, W. et al. Transcriptome dynamics of hippocampal neurogenesis in macaques across the lifespan and aged humans. *Cell Res.*10.1038/s41422-022-00678-y (2022). 10.1038/s41422-022-00678-y PMC9343414 35750757

[50] Tosoni G (2023). Mapping human adult hippocampal neurogenesis with single-cell transcriptomics: reconciling controversy or fueling the debate?. Neuron.

[51] Murakawa R, Kosaka T (1999). Diversity of the calretinin immunoreactivity in the dentate gyrus of gerbils, hamsters, guinea pigs, and laboratory shrews. J. Comp. Neurol.

[52] Kozorovitskiy Y, Gould E (2004). Dominance hierarchy influences adult neurogenesis in the dentate gyrus. J. Neurosci..

[53] Lagace DC, Fischer SJ, Eisch AJ (2007). Gender and endogenous levels of estradiol do not influence adult hippocampal neurogenesis in mice. Hippocampus.

[54] Llorens-Martin M (2013). GSK-3beta overexpression causes reversible alterations on postsynaptic densities and dendritic morphology of hippocampal granule neurons in vivo. Mol. Psychiatry.

[55] Bonzano S (2018). Neuron-astroglia cell fate decision in the adult mouse hippocampal neurogenic niche Is cell-intrinsically controlled by COUP-TFI in vivo. Cell Rep..

[56] Janssens J (2019). Evaluating the applicability of mouse SINEs as an alternative normalization approach for RT-qPCR in brain tissue of the APP23 model for Alzheimer’s disease. J. Neurosci. Methods.

[57] Llorens-Martin M, Torres-Aleman I, Trejo JL (2006). Pronounced individual variation in the response to the stimulatory action of exercise on immature hippocampal neurons. Hippocampus.

