# Supplementary material for: Prolonged fixation and post-mortem delay impede the study of adult neurogenesis in mice
Source: Commun Biol. 2023 Sep 23;6:978. doi: 10.1038/s42003-023-05367-z (PMC10517969; doi:10.1038/s42003-023-05367-z)
Supplement: Supplementary file 1 — Supplementary material [file 42003_2023_5367_MOESM1_ESM.pdf]

Supplementary Material for

"Prolonged fixation and post-mortem delay impede the study of adult neurogenesis in mice"

M. Gallardo-Caballero[a,b,c]; C.B. Rodríguez-Moreno[a,b]; L. Álvarez-Méndez[a,c]; J. Terreros-Roncal[a,b]; M. Flor-García[a,b,c]; E.P. Moreno-Jiménez[a,b,c]; A. Rábano[d]; M. Llorens-Martín[a,b,c]*.

[a] Department of Molecular Neuropathology, Centro de Biología Molecular "Severo Ochoa" (CBMSO), Spanish Research Council (CSIC)–Universidad Autónoma de Madrid (UAM); Madrid (Spain).

[b] Center for Networked Biomedical Research on Neurodegenerative Diseases (CIBERNED); Madrid (Spain).

[c] Department of Molecular Biology, Faculty of Sciences, Universidad Autónoma de Madrid; Madrid (Spain).

[d] CIEN Foundation; Madrid (Spain).

*Correspondence should be addressed to: **María Llorens-Martín** (m.llorens@csic.es, Centro de Biología Molecular "Severo Ochoa", Spanish Research Council (CSIC). Universidad Autónoma de Madrid (campus de Cantoblanco), c/Nicolás Cabrera 1, 28049, Madrid (Spain). Phone number: +34-911964632.

Running title: Prolonged fixation and post-mortem delay impede the study of adult neurogenesis

Keywords: Adult hippocampal neurogenesis; post-mortem delay, fixation, doublecortin

Supplementary Figure 1: Impact of the post-mortem delay (PMD) and fixation time on the detection of markers of immature dentate granule cells (DGCs).

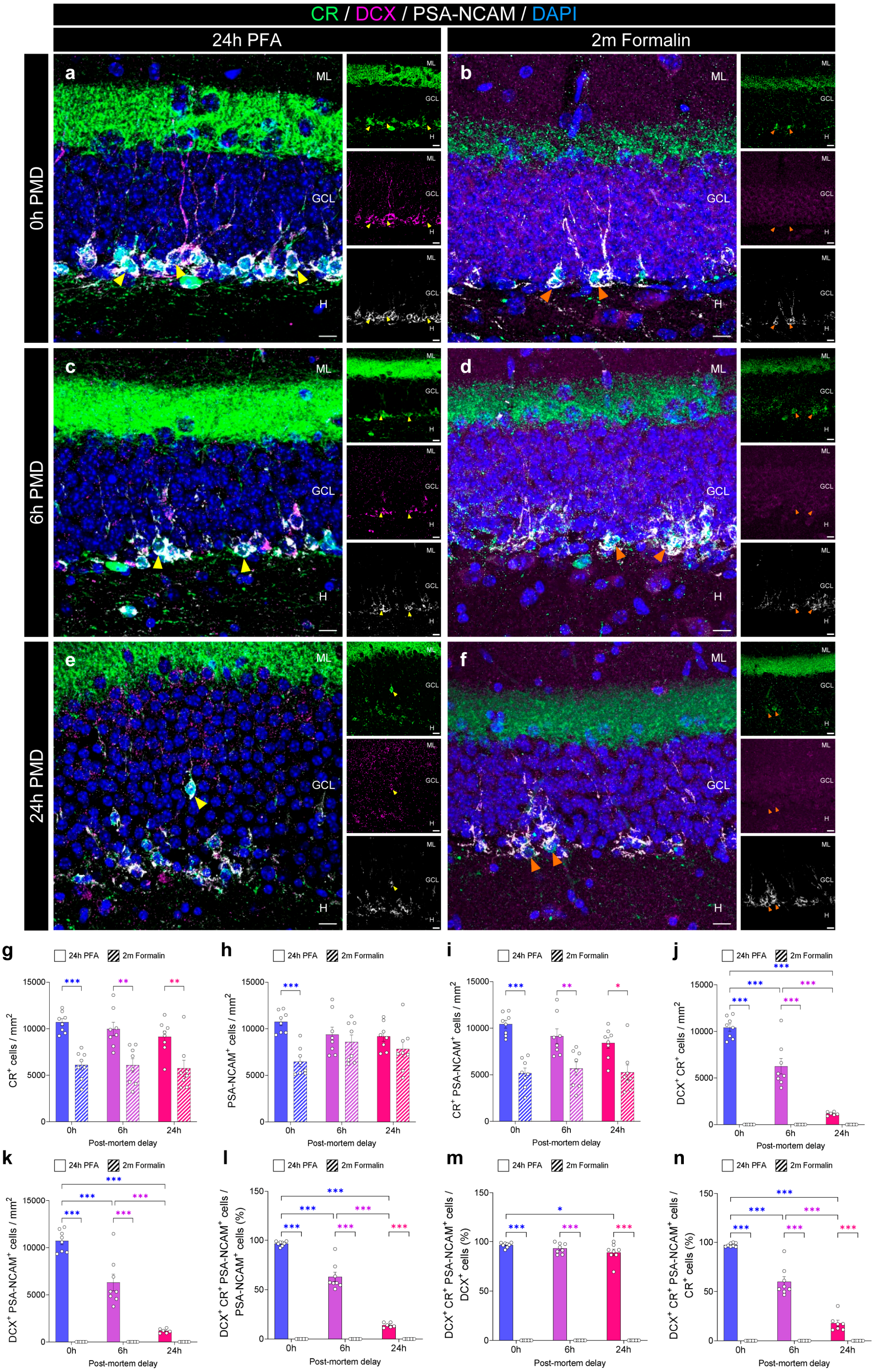

a – f: Representative images of Doublecortin $(DCX)^+$, Calretinin $(CR)^+$, and Polysialylated-neural cell adhesion molecule $(PSA\text{-}NCAM)^+$ staining under distinct experimental conditions. The DCX and DAPI images displayed are the same as those included in **Figure 1 b – g**. In this supplementary figure, the CR and PSA-NCAM channels have been added and merged images are shown. **g:** Density of CR^+ immature DGCs. **h:** Density of $PSA\text{-}NCAM^+$ immature DGCs. **i:** Density of CR^+ $PSA\text{-}NCAM^+$ immature DGCs. **j:** Density of DCX^+ CR^+ immature DGCs. **k:** Density of DCX^+ $PSA\text{-}NCAM^+$ immature DGCs. **l:** Percentage of DCX^+ CR^+ $PSA\text{-}NCAM^+$ out of total $PSA\text{-}NCAM^+$ cells. **m:** Percentage of DCX^+ CR^+ $PSA\text{-}NCAM^+$ out of total DCX^+ cells. **n:** Percentage of DCX^+ CR^+ $PSA\text{-}NCAM^+$ out of total CR^+ cells. ML: Molecular layer. GCL: Granule cell layer. H: Hilus. White scale bar: 10 μm. Yellow triangles: DCX^+ CR^+ $PSA\text{-}NCAM^+$ immature DGCs. Orange triangles: CR^+ $PSA\text{-}NCAM^+$ immature DGCs. Graphs represent mean values ± SEM. n = 8 mice. * $0.05 > p \geq 0.01$; ** $0.01 > p \geq 0.001$; and *** $p < 0.001$.

Supplementary Figure 2: Performance of distinct anti-Doublecortin (DCX) antibodies under distinct experimental conditions.

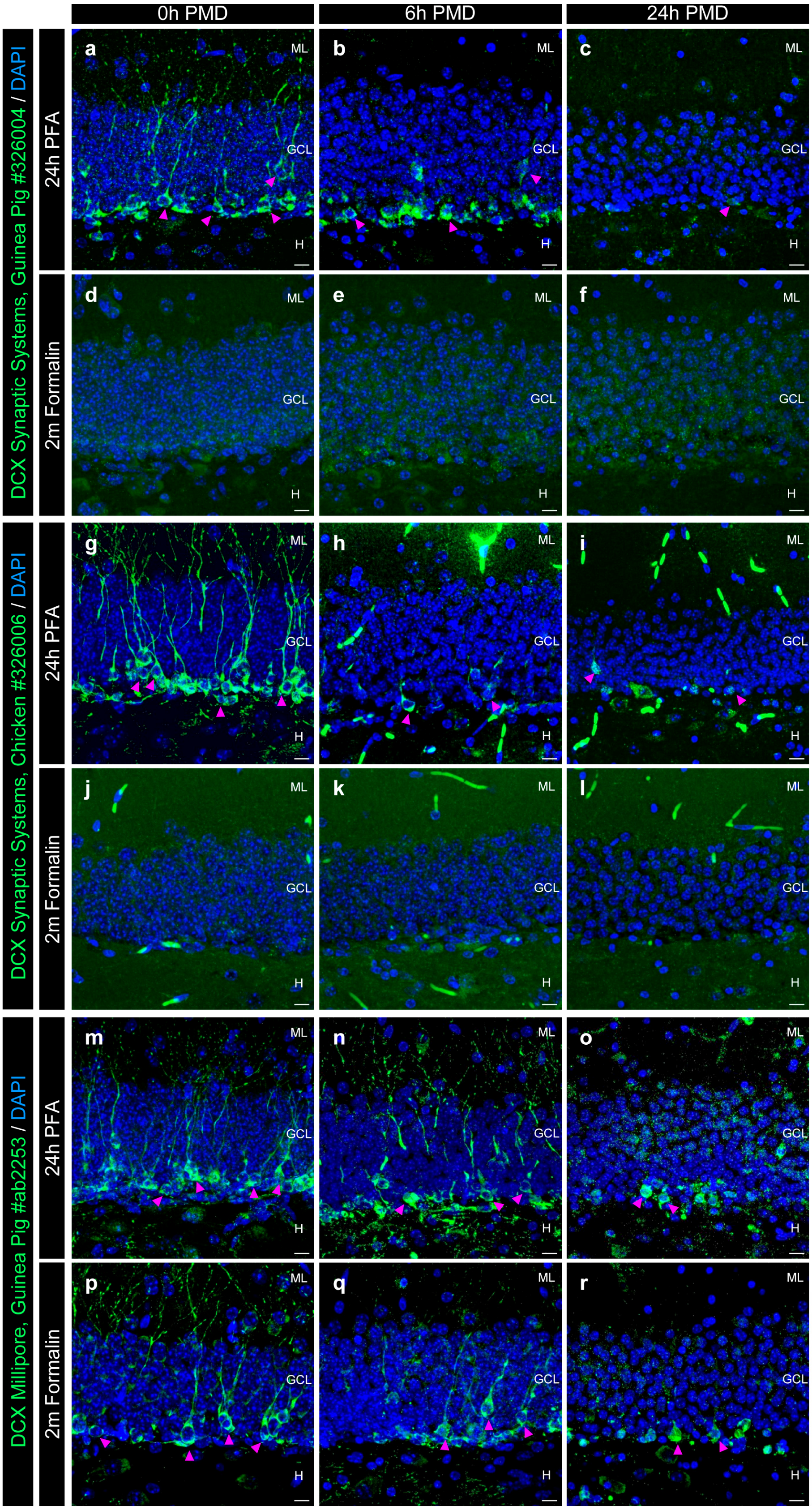

a – r: Representative images of DCX staining using a guinea pig (**a** – **f**: Synaptic Systems #326004), a chicken (**g** – **l**: Synaptic systems #326006), and another guinea pig (**m** – **r**: Milliepore #ab2253) anti-DCX antibody. Z-projection images are shown. ML: Molecular layer. GCL: Granule cell layer. H: Hilus. White scale bar: 10 μm. Magenta triangles: DCX^+ immature DGCs.

Supplementary Figure 3: Performance of distinct anti-Doublecortin (DCX) antibodies under distinct experimental conditions.

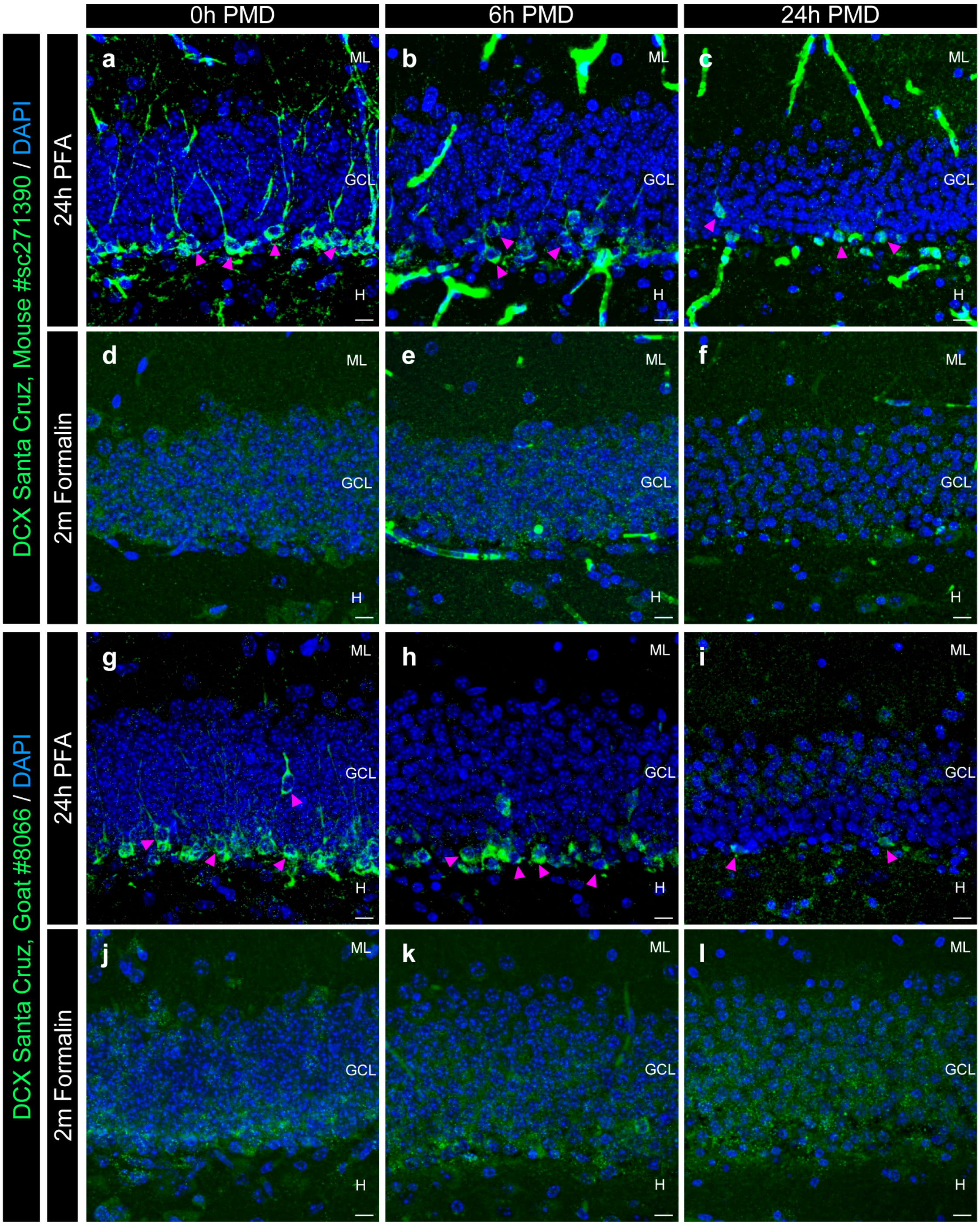

a – r: Representative images of DCX staining using a mouse (**a – f**: Santa Cruz #sc271390) and a goat (**g – l**: Santa Cruz #8066) anti-DCX antibodies. Z-projection images are shown. ML: Molecular layer. GCL: Granule cell layer. H: Hilus. White scale bar: 10 μm. Magenta triangles: DCX^+ immature DGCs.

Supplementary Figure 4: Performance of distinct rabbit anti-Doublecortin (DCX) antibodies under distinct experimental conditions.

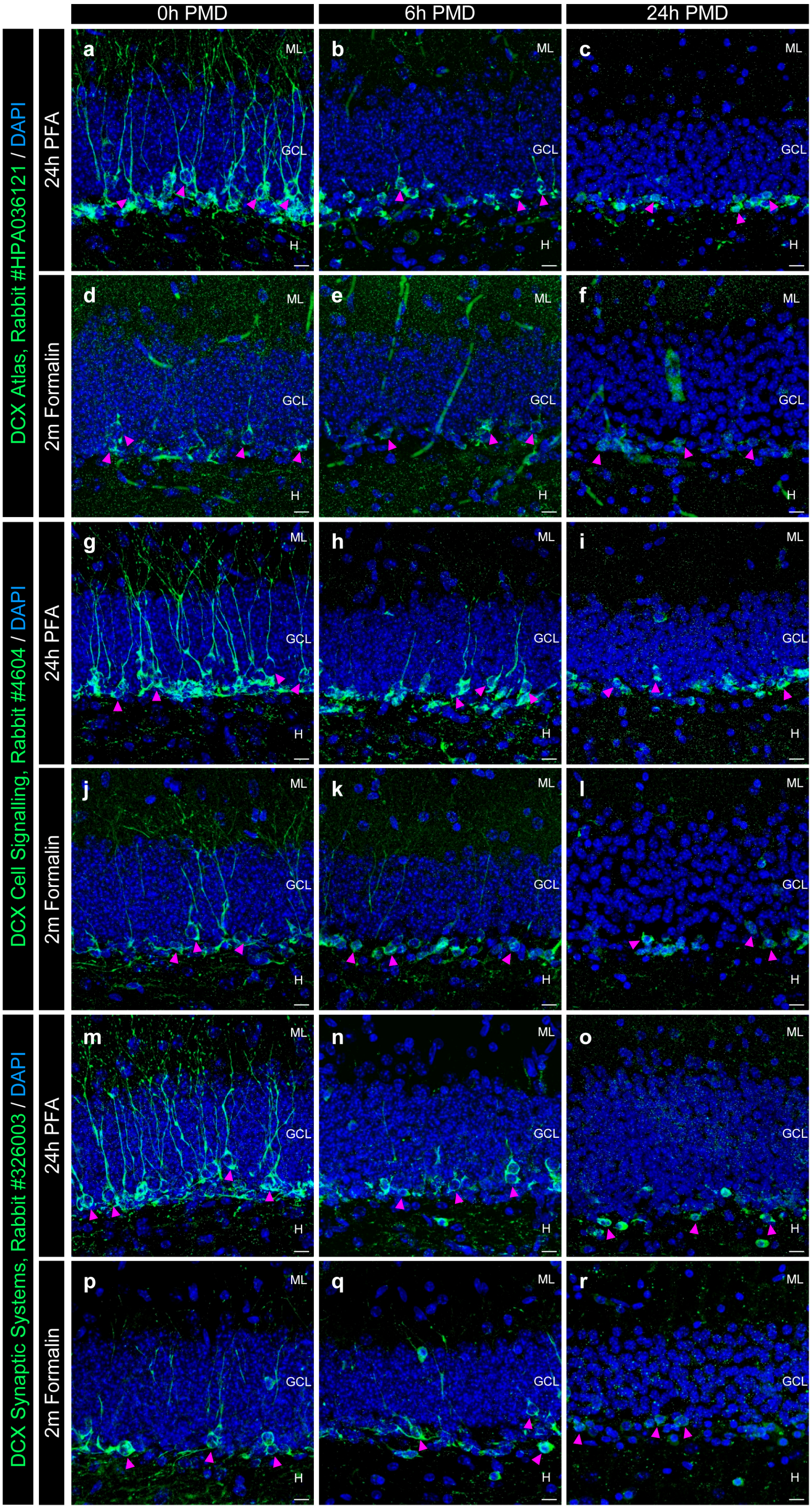

a – r: Representative images of DCX staining using three rabbit anti-DCX antibodies (**a** – **f**: Atlas Antibodies #HPA036121; **g** – **l**: Cell Signalling #4604; **m** – **r**: Synaptic systems #326003). Z-projection images are shown. ML: Molecular layer. GCL: Granule cell layer. H: Hilus. White scale bar: 10 μm. Magenta triangles: DCX^+ immature DGCs.

Supplementary Figure 5: Performance of distinct rabbit anti-Doublecortin (DCX) antibodies under distinct experimental conditions.

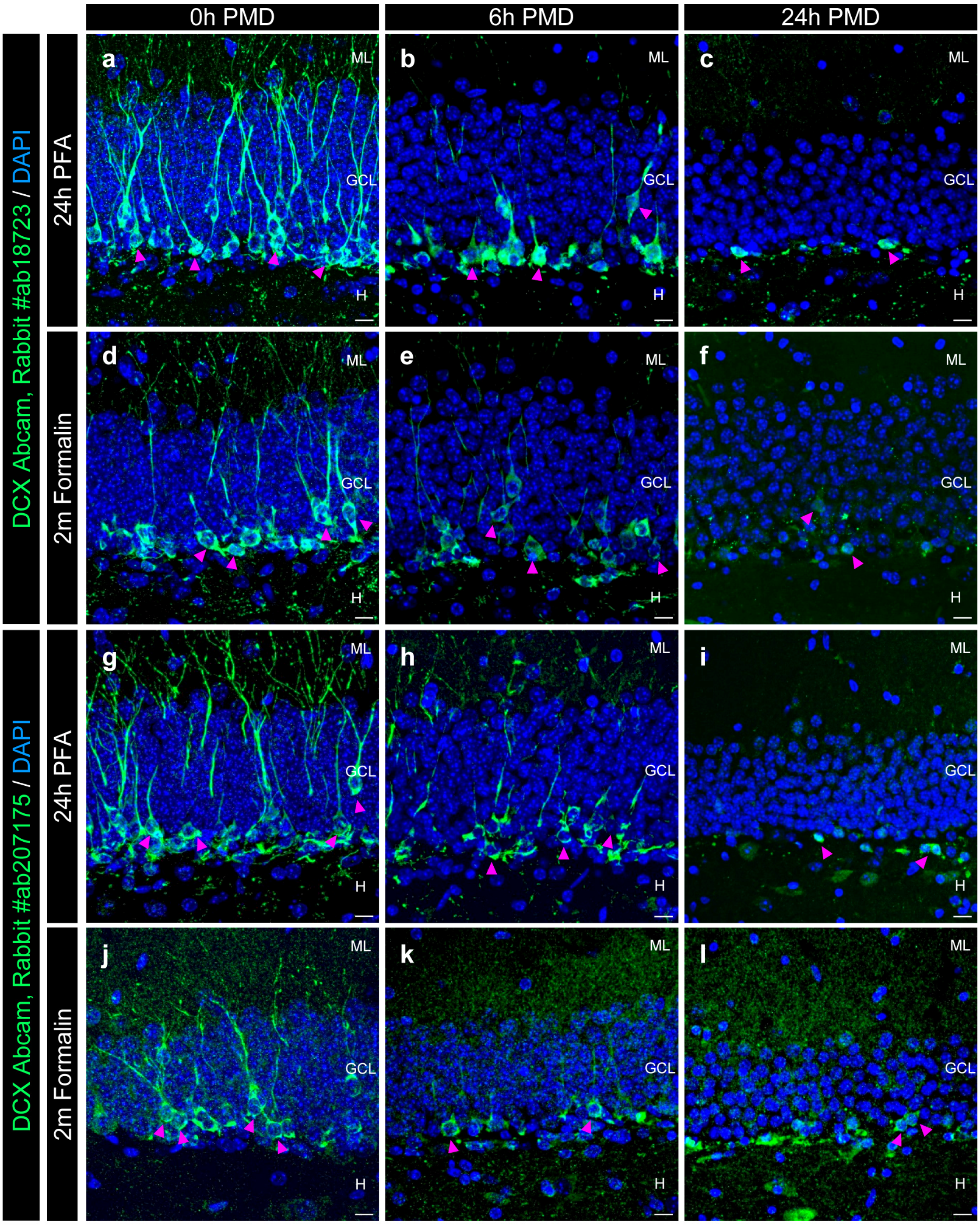

a – l: Representative images of DCX staining using two rabbit anti-DCX antibodies (**a – f**: Abcam #ab18723; **g – l**: Abcam #ab207175). Z-projection images are shown. ML: Molecular layer. GCL: Granule cell layer. H: Hilus. White scale bar: 10 µm. Magenta triangles: DCX^+ immature DGCs.

Supplementary Figure 6: Impact of the post-mortem delay (PMD) and fixation time on the detection of Ki67$^+$ proliferative cells.

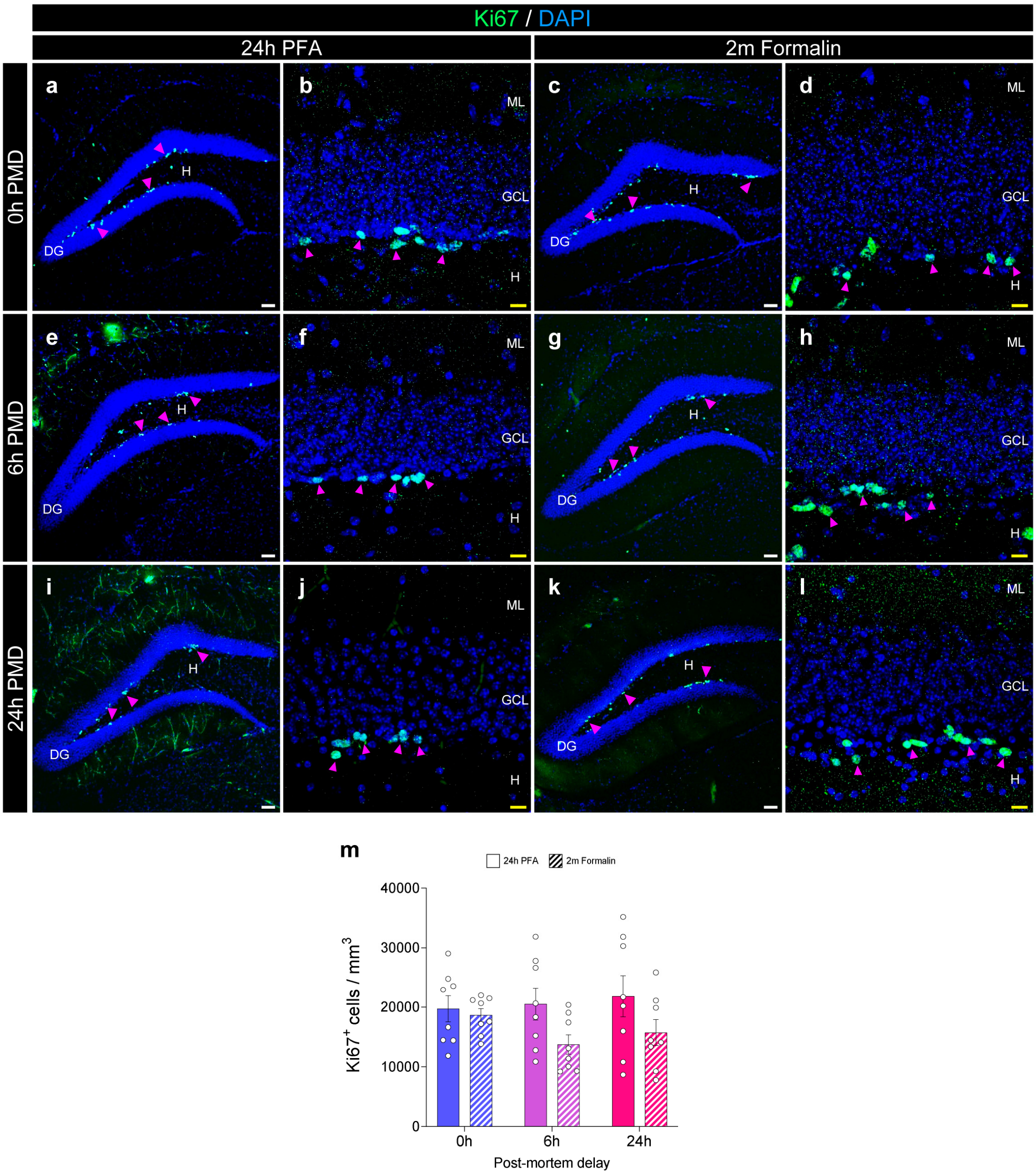

a – l: Representative images of Ki67 staining under distinct experimental conditions. **m:** Density of Ki67$^+$ proliferative cells. In **a – l**, Z-projection images are shown. DG: Dentate Gyrus. ML: Molecular layer. GCL: Granule cell layer. H: Hilus. White scale bar: 50 µm. Yellow scale bar: 10 µm. Magenta triangles: Ki67$^+$ cells. Graphs represent mean values ± SEM. n = 8 mice.

Supplementary Figure 7: Performance of distinct anti-Doublecortin (DCX) antibodies after 0.5% $NaBH_4$ and heat-mediated citrate buffer antigen retrieval (HC-AR) pre-treatments on samples subjected to 0 h post-mortem delay and prolonged fixation.

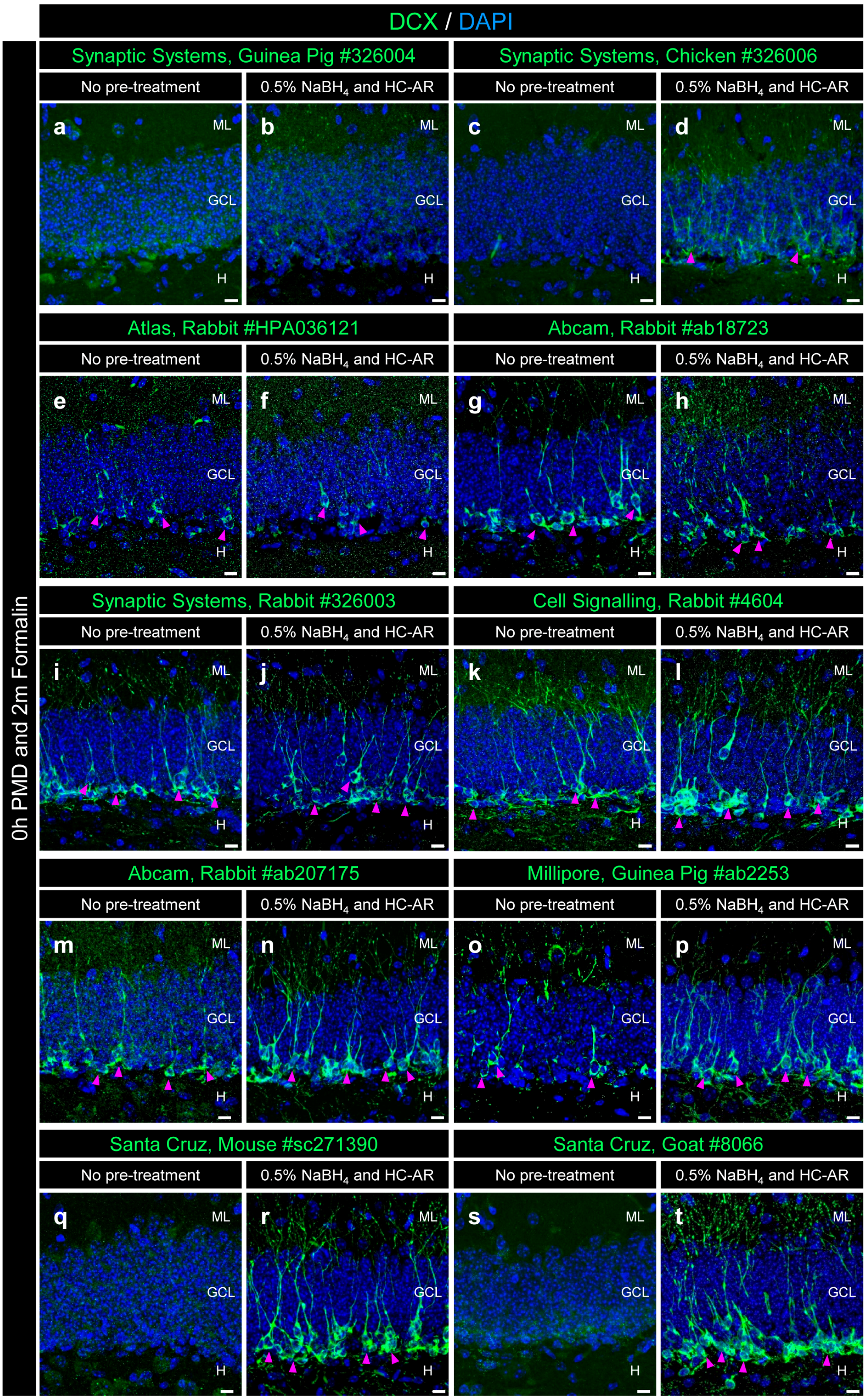

a – t: Representative images of DCX staining using distinct antibodies (**a – b:** Synaptic Systems, Guinea Pig #326004; **c – d:** Synaptic Systems, Chicken #326006; **e – f:** Atlas, Rabbit #HPA036121; **g – h:** Abcam, Rabbit #ab18723; **i – j:** Synaptic Systems, Rabbit #326003; **k – l:** Cell Signalling, Rabbit #4604; **m – n:** Abcam, Rabbit #ab207175; **o – p:** Millipore, Guinea Pig #ab2253; **q – r:** Santa Cruz, Mouse #sc271390; **s – t:** Santa Cruz, Goat #8066). Z-projection images are shown. ML: Molecular layer. GCL: Granule cell layer. H: Hilus. White scale bar: 10 μm. Magenta triangles: DCX+ immature DGCs.

Supplementary Figure 8: Impact of the method of euthanasia on the detection of Doublecortin (DCX)$^+$ cells.

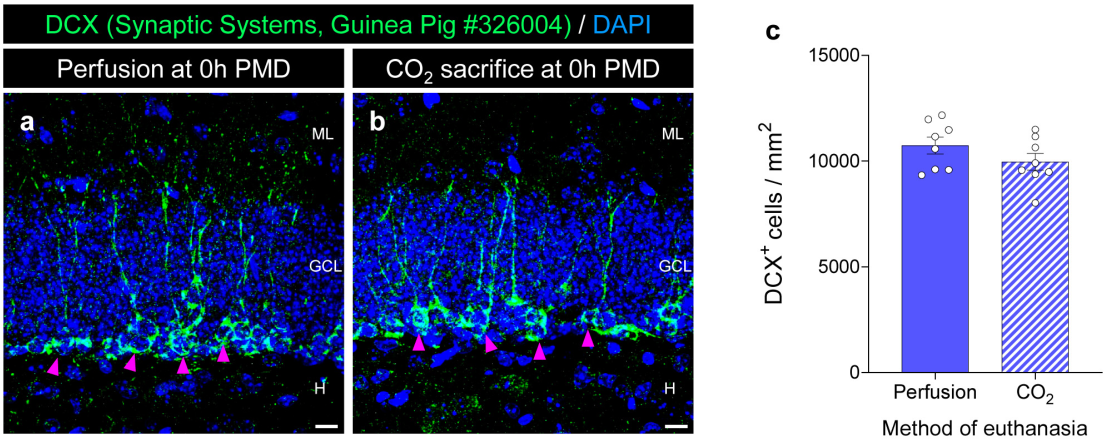

a – b: Representative images of anti-DCX (Synaptic Systems, Guinea Pig #326004#) staining under distinct experimental conditions. **c:** Density of DCX$^+$ immature dentate granule cells detected. In **a – b**, Z-projection images are shown. ML: Molecular layer. GCL: Granule cell layer. H: Hilus. White scale bar: 10 µm. Magenta triangles: DCX$^+$ immature DGCs. Graphs represent mean values ± SEM. n = 8 mice.

Supplementary Figure 9: Raw Western blot image.

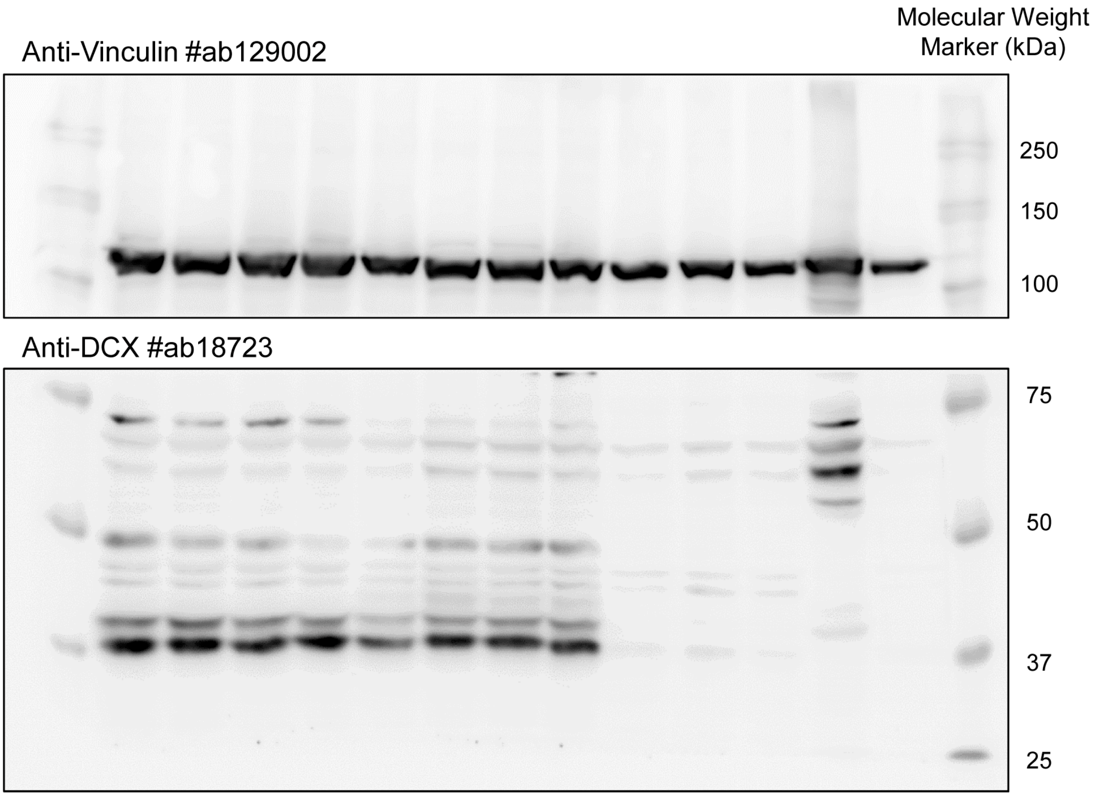

Supplementary Table 1. List of primary antibodies used.

Antibody	Species	Company	Catalog Number	RRID	Working concentration	Use
Calbindin (CB)	Mouse	Sigma	#C9848	AB_476894	1/400	IHC
Calretinin (CR)	Rabbit	Swant	#CR-7697	AB_2619710	1/500	IHC
Doublecortin (DCX)	Chicken	Synaptic Systems	#326006	AB_2737040	1/500	IHC
Doublecortin (DCX)	Goat	Santa Cruz	#8066 (discontinued)	AB_2088494	1/1,000	IHC
Doublecortin (DCX)	Guinea Pig	Millipore	#ab2253	AB_1586992	1/250	IHC
Doublecortin (DCX)	Guinea Pig	Synaptic Systems	#326004	AB_2620068	1/500	IHC
Doublecortin (DCX)	Mouse	Santa Cruz	#sc271390	AB_10610966	1/50	IHC
Doublecortin (DCX)	Rabbit	Abcam	#ab18723	AB_732011	1/500	IHC and WB
Doublecortin (DCX)	Rabbit	Atlas	#HPA036121	AB_2674950	1/100	IHC
Doublecortin (DCX)	Rabbit	Cell Signalling	#4604	AB_561007	1/250	IHC
Doublecortin (DCX)	Rabbit	Synaptic Systems	#326003	AB_2620067	1/500	IHC
Doublecortin (DCX)	Rabbit	Abcam	#ab207175	AB_2894710	1/1,000	IHC
Iba1	Chicken	Synaptic Systems	#234006 (discontinued)	AB_2619949	1/1,000	IHC
Ki67	Rabbit	Abcam	#ab15580	AB_443209	1/500	IHC
Polysialylated neural cell adhesion molecule (PSA-NCAM)	Mouse	Millipore	#mab5324	AB_95211	1/1,000	IHC
S100 calcium-binding protein β (S100β)	Guinea Pig	Synaptic Systems	#287004	AB_2620025	1/500	IHC
SRY (sex determining region Y)-box 2 (Sox2)	Goat	R and D Systems	#AF2018	AB_355110	1/700	IHC
Vimentin	Rabbit	Abcam	#ab193555	AB_2814713	1/1,000	IHC
Vinculin	Rabbit	Abcam	#ab129002	AB_11144129	1/7,500	WB

The antigen, host species, company, catalog number, RRID, concentration, and use are shown.

Supplementary Table 2. List of secondary antibodies used.

Antibody	Species	Company	Catalog Number	RRID	Working concentration	Use
Alexa-488 Anti-Rabbit	Donkey	Thermo Fisher	#A-21206	AB_2535792	1/1,000	IHC
Alexa-555 Anti-Mouse	Donkey	Thermo Fisher	#A-31570	AB_2536180	1/1,000	IHC
Alexa-555 Anti-Guinea Pig	Goat	Thermo Fisher	#A-21435	AB_2535856	1/1,000	IHC
Alexa-555 Anti-Goat	Donkey	Thermo Fisher	#A-21432	AB_2535853	1/1,000	IHC
Alexa-647 Anti-Mouse	Donkey	Thermo Fisher	#A-31571	AB_162542	1/1,000	IHC
Alexa-647 Anti-Guinea Pig	Donkey	Jackson ImmunoResearch	#706-605-148	AB_2340476	1/1,000	IHC
Alexa-647 Anti-Chicken	Goat	Thermo Fisher	#A-21449	AB_2535866	1/1,000	IHC
HRP Anti-Rabbit	Goat	Dako	#P0448	AB_2617138	1/1,000	WB

The antigen, host species, company, catalog number, RRID, concentration, and use are shown.
