# Supplementary material for: Prolonged fixation and post-mortem delay impede the study of adult neurogenesis in mice
Source: Commun Biol. 2023 Sep 23;6:978. doi: 10.1038/s42003-023-05367-z (PMC10517969; doi:10.1038/s42003-023-05367-z)
Supplement: Supplementary file 2 — Description of Additional Supplementary Files [file 42003_2023_5367_MOESM2_ESM.pdf]

Description of Additional Supplementary Files

File name: Supplementary Data 1
Description: Detailed results from statistical comparisons. The results of all statistical comparisons included in this manuscript are presented. Each tab includes all the statistical comparisons that refer to a single Figure or Supplementary Figure.

File name: Supplementary Data 2
Description: Numerical source data. Source data underlying the graphs and charts presented in the Main and Supplementary Figures.
