# Supplementary material for: Prolonged fixation and post-mortem delay impede the study of adult neurogenesis in mice
Source: Commun Biol. 2023 Sep 23;6:978. doi: 10.1038/s42003-023-05367-z (PMC10517969; doi:10.1038/s42003-023-05367-z)
Supplement: Supplementary file 4 — Reporting Summary [file 42003_2023_5367_MOESM4_ESM.pdf]

Corresponding author(s): MARÍA LLORENS-MARTÍN
Last updated by author(s): Apr 26, 2023

Reporting Summary

Nature Portfolio wishes to improve the reproducibility of the work that we publish. This form provides structure for consistency and transparency in reporting. For further information on Nature Portfolio policies, see our Editorial Policies and the Editorial Policy Checklist.

Statistics

For all statistical analyses, confirm that the following items are present in the figure legend, table legend, main text, or Methods section.

n/a	Confirmed	
☐	☒	The exact sample size (*n*) for each experimental group/condition, given as a discrete number and unit of measurement
☐	☒	A statement on whether measurements were taken from distinct samples or whether the same sample was measured repeatedly
☐	☒	The statistical test(s) used AND whether they are one- or two-sided<br>*Only common tests should be described solely by name; describe more complex techniques in the Methods section.*
☐	☒	A description of all covariates tested
☐	☒	A description of any assumptions or corrections, such as tests of normality and adjustment for multiple comparisons
☐	☒	A full description of the statistical parameters including central tendency (e.g. means) or other basic estimates (e.g. regression coefficient) AND variation (e.g. standard deviation) or associated estimates of uncertainty (e.g. confidence intervals)
☐	☒	For null hypothesis testing, the test statistic (e.g. F, t, r) with confidence intervals, effect sizes, degrees of freedom and P value noted<br>*Give P values as exact values whenever suitable.*
☒	☐	For Bayesian analysis, information on the choice of priors and Markov chain Monte Carlo settings
☒	☐	For hierarchical and complex designs, identification of the appropriate level for tests and full reporting of outcomes
☒	☐	Estimates of effect sizes (e.g. Cohen's *d*, Pearson's *r*), indicating how they were calculated

Our web collection on statistics for biologists contains articles on many of the points above.

Software and code

Policy information about availability of computer code

Data collection	Fiji (ImageJ 64 bits 1.53t) was used to analyze confocal images
Data analysis	Data were analyzed using GraphPad Prism 9 software (GraphPad.v.9.4.1 (681) 2022; GraphPad Software, LLC).

For manuscripts utilizing custom algorithms or software that are central to the research but not yet described in published literature, software must be made available to editors and reviewers. We strongly encourage code deposition in a community repository (e.g. GitHub). See the Nature Portfolio guidelines for submitting code & software for further information.

Data

Policy information about availability of data

All manuscripts must include a data availability statement. This statement should provide the following information, where applicable:

- Accession codes, unique identifiers, or web links for publicly available datasets
- A description of any restrictions on data availability
- For clinical datasets or third party data, please ensure that the statement adheres to our policy

All the materials are available from the corresponding author upon reasonable request

Research involving human participants, their data, or biological material

Policy information about studies with human participants or human data. See also policy information about sex, gender (identity/presentation), and sexual orientation and race, ethnicity and racism.

Reporting on sex and gender	No human subjects participate in this study
Reporting on race, ethnicity, or other socially relevant groupings	N/A
Population characteristics	N/A
Recruitment	N/A
Ethics oversight	N/A

Note that full information on the approval of the study protocol must also be provided in the manuscript.

Field-specific reporting

Please select the one below that is the best fit for your research. If you are not sure, read the appropriate sections before making your selection.

☒ Life sciences ☐ Behavioural & social sciences ☐ Ecological, evolutionary & environmental sciences

For a reference copy of the document with all sections, see nature.com/documents/nr-reporting-summary-flat.pdf

Life sciences study design

All studies must disclose on these points even when the disclosure is negative.

Sample size	Sample size was determined according to our knowledge from previous studies.
Data exclusions	Atypical data were identified using using GraphPad Prism 9 software (GraphPad.v.9.4.1 (681), 2022; GraphPad Software, LLC) and extreme outlier values were eliminated when necessary.
Replication	The number of biological replicates is detailed in the Material and Methods subsections
Randomization	Mice were randomly allocated to the distinct experimental groups
Blinding	No specific blinding strategy was used

Reporting for specific materials, systems and methods

We require information from authors about some types of materials, experimental systems and methods used in many studies. Here, indicate whether each material, system or method listed is relevant to your study. If you are not sure if a list item applies to your research, read the appropriate section before selecting a response.

Materials & experimental systems

n/a	Involved in the study	
☐	☒	Antibodies
☐	☐	Eukaryotic cell lines
☐	☐	Palaeontology and archaeology
☐	☒	Animals and other organisms
☐	☐	Clinical data
☐	☐	Dual use research of concern
☐	☐	Plants

Methods

n/a	Involved in the study	
☐	☐	ChIP-seq
☐	☐	Flow cytometry
☐	☐	MRI-based neuroimaging

Antibodies

Antibodies used	The following primary antibodies have been used in immunofluorescence:<br>Mouse anti-Calbindin, Sigma #C9848, RRID: AB_476894, 1/400<br>Rabbit anti-Calretinin, Swant #CR-7697, RRID: AB_2619710, 1/500

Chicken anti-Doublecortin, Synaptic Systems #326006, RRID: AB_2737040, 1/500
Goat anti-Doublecortin, Santa Cruz #8066 (discontinued), RRID: AB_2088494, 1/1,000
Guinea Pig anti-Doublecortin, Millipore #ab2253, RRID: AB_1586992, 1/250
Guinea Pig anti-Doublecortin, Synaptic Systems #326004, RRID: AB_2620068, 1/500
Mouse anti-Doublecortin, Santa Cruz #sc271390, RRID: AB_10610966, 1/50
Rabbit anti-Doublecortin, Abcam #ab18723, RRID: AB_732011, 1/500
Rabbit anti-Doublecortin, Atlas #HPA036121, RRID: AB_2674950, 1/100
Rabbit anti-Doublecortin, Cell Signalling #4604, RRID: AB_561007, 1/250
Rabbit anti-Doublecortin, Synaptic Systems #326003, RRID:AB_2620067, 1/500
Rabbit anti-Doublecortin, Abcam #ab207175, RRID: AB_2894710, 1/1,000
Chicken anti-Iba1, Synaptic Systems #234006 (discontinued), RRID: AB_2619949, 1/1,000
Rabbit anti-Ki67, Abcam #ab15580, RRID: AB_443209, 1/500
Mouse anti- Polysialylated neural cell adhesion molecule, Millipore #mab5324, RRID: AB_95211, 1/1,000
Guinea pig anti-S100 calcium-binding protein β, Synaptic Systems #287004, RRID: AB_2620025, 1/500
Goat anti-SRY (sex determining region Y)-box 2 (Sox2), R and D Systems #AF2018, RRID: AB_355110, 1/700
Rabbit anti-Vimentin, Abcam #ab193555, RRID: AB_2814713, 1/1,000
Rabbit anti-Vinculin, Abcam #ab129002, RRID:AB_11144129, 1/7,500

The following secondary antibodies were used to detect the binding of primary antibodies:
Donkey Alexa-488 Anti-Rabbit, Thermo Fisher #A-21206, RRID: AB_2535792, 1/1,000
Donkey Alexa-555 Anti-Mouse, Thermo Fisher #A-31570, RRID: AB_2536180, 1/1,000
Goat Alexa-555 Anti-Guinea Pig , Thermo Fisher #A-21435, RRID: AB_2535856, 1/1,000
Donkey Alexa-555 Anti-Goat, Thermo Fisher #A-21432, RRID: AB_2535853, 1/1,000
Donkey Alexa-647 Anti-Mouse, Thermo Fisher #A-31571, RRID: AB_162542, 1/1,000
Donkey Alexa-647 Anti-Guinea Pig, Jackson ImmunoResearch #706-605-148, RRID: AB_2340476, 1/1,000
Goat Alexa-647 Anti-Chicken, Thermo Fisher #A-21449, RRID: AB_2535866, 1/1,000
Goat HRP Anti-Rabbit, Dako #P0448, RRID: AB_2617138, 1/1,000

Validation

- Mouse anti-Calbindin, Sigma #C9848, RRID: AB_476894, 1/400: Validated by manufacturer to detect Calbindin of mouse, human, guinea pig, canine, sheep, rat, pig, rabbit, feline, bovine, goat origin by IHC, WB and ELISA.

- Rabbit anti-Calretinin, Swant #CR-7697, RRID: AB_2619710, 1/500: validated by manufacturer to detect Calretinin of human, monkey, rat, mouse, guinea pig, chicken, and fish origin by WB and IHC.

- Chicken anti-Doublecortin, Synaptic Systems #326006, RRID: AB_2737040, 1/500: validated by manufacturer to detect Doublecortin of mice origin by ICC and IHC.

- Goat anti-Doublecortin, Santa Cruz #8066 (discontinued), RRID: AB_2088494, 1/1,000: Validated by manufacturer to detect Doublecortin of mouse, rat, human, and avian origin by WB, IP, IF, IHC, and ELISA.

- Guinea Pig anti-Doublecortin, Millipore #ab2253, RRID: AB_1586992, 1/250: Validated by manufacturer to detect Doublecortin of mouse and rat origin by WB, IHC and ICC.

- Guinea Pig anti-Doublecortin, Synaptic Systems #326004, RRID: AB_2620068, 1/500: validated by manufacturer to detect Doublecortin of mice and rat origin by WB, IP, ICC and IHC.

- Mouse anti-Doublecortin, Santa Cruz #sc271390, RRID: AB_10610966, 1/50: Validated by manufacturer to detect Doublecortin of mouse, rat, and human origin by WB, IP, IF, IHC, and ELISA.

- Rabbit anti-Doublecortin, Abcam #ab18723, RRID: AB_732011, 1/500: Validated by manufacturer to detect Doublecortin of mouse, rat, and quail origin by WB, IHC and ICC.

- Rabbit anti-Doublecortin, Atlas #HPA036121, RRID: AB_2674950, 1/100: Validated by manufacturer to detect Doublecoritn of human origin by IHC

- Rabbit anti-Doublecortin, Cell Signalling #4604, RRID: AB_561007, 1/250: Validated by manufacturer to detect Doublecortin of mouse and rat origin by WB, IP, IF, IHC and ICC.

- Rabbit anti-Doublecortin, Synaptic Systems #326003, RRID: AB_2620067, 1/500: validated by manufacturer to detect Doublecortin of mice, rat and human origin by WB, IP, ICC and IHC.

- Rabbit anti-Doublecortin, Abcam #ab207175, RRID: AB_2894710, 1/1,000: validated by manufacturer to detect Doublecortin of mice, rat and human origin by WB, IF, ICC and IHC.

- Chicken anti-Iba1, Synaptic Systems #234006 (discontinued), RRID: AB_2619949, 1/1,000: validated by manufacturer to detect Iba1 of mice, rat and human origin by WB, IF, ICC and IHC.

- Rabbit anti-Ki67, Abcam #ab15580, RRID: AB_443209, 1/500: validated by manufacturer to detect Ki-67 of mice and human origin by ICC and IHC.

- Mouse anti- Polysialylated neural cell adhesion molecule (PSA-NCAM), Millipore #mab5324, RRID: AB_95211, 1/1,000: validated by manufacturer to detect PSA-NCAM of mice, rat and human origin by ICC, IHC, RIA and WB.

- Guinea pig anti-S100 calcium-binding protein β (S100β), Synaptic Systems #287004, RRID: AB_2620025, 1/500: validated by

manufacturer to detect S100β of mice and rat origin by ICC and IHC.

- Goat anti-SRY (sex determining region Y)-box 2 (Sox2), R and D Systems #AF2018, RRID: AB_355110, 1/700: validated by manufacturer to detect Sox2 of mice, rat and human origin by WB, ChIP and ICC.

- Rabbit anti-Vimentin, Abcam #ab193555, RRID: AB_2814713, 1/1,000: validated by manufacturer to detect Vimentin of mice, rat, African green monkey and human origin by WB, Flow cytometry, IHC, IF and ICC.

- Rabbit anti-Vinculin, Abcam #ab129002, RRID:AB_11144129, 1/7,500: validated by manufacturer to detect Vinculin of mice, rat and human origin by WB, Flow cytometry, IP, IF and ICC.

Secondary antibodies were validated by manufacturer and have been extensively validated in the literature.

Eukaryotic cell lines

Policy information about cell lines and Sex and Gender in Research

Cell line source(s)	N/A
Authentication	N/A
Mycoplasma contamination	N/A
Commonly misidentified lines (See ICLAC register)	N/A

Palaeontology and Archaeology

Specimen provenance	N/A
Specimen deposition	N/A
Dating methods	N/A

☐ Tick this box to confirm that the raw and calibrated dates are available in the paper or in Supplementary Information.

Ethics oversight	N/A

Note that full information on the approval of the study protocol must also be provided in the manuscript.

Animals and other research organisms

Policy information about studies involving animals; ARRIVE guidelines recommended for reporting animal research, and Sex and Gender in Research

Laboratory animals	Seven- to nine-week-old C57BL/6J-OlaHsd mice
Wild animals	N/A
Reporting on sex	All mice included in the study were female.
Field-collected samples	N/A
Ethics oversight	Animal experiments were approved by the CBMSO (AEEC-CBMSO-23/172) and National (PROEX 185.4/20) Ethics Committees.

Note that full information on the approval of the study protocol must also be provided in the manuscript.

Clinical data

Policy information about clinical studies

All manuscripts should comply with the ICMJE guidelines for publication of clinical research and a completed CONSORT checklist must be included with all submissions.

Clinical trial registration	N/A
Study protocol	N/A
Data collection	N/A

Outcomes	N/A

Dual use research of concern

Policy information about dual use research of concern

Hazards

Could the accidental, deliberate or reckless misuse of agents or technologies generated in the work, or the application of information presented in the manuscript, pose a threat to:

No	Yes	
☒	☐	Public health
☒	☐	National security
☒	☐	Crops and/or livestock
☒	☐	Ecosystems
☒	☐	Any other significant area

Experiments of concern

Does the work involve any of these experiments of concern:

No	Yes	
☒	☐	Demonstrate how to render a vaccine ineffective
☒	☐	Confer resistance to therapeutically useful antibiotics or antiviral agents
☒	☐	Enhance the virulence of a pathogen or render a nonpathogen virulent
☒	☐	Increase transmissibility of a pathogen
☒	☐	Alter the host range of a pathogen
☒	☐	Enable evasion of diagnostic/detection modalities
☒	☐	Enable the weaponization of a biological agent or toxin
☒	☐	Any other potentially harmful combination of experiments and agents

Plants

Seed stocks	N/A
Novel plant genotypes	N/A
Authentication	N/A

ChIP-seq

Data deposition

☐ Confirm that both raw and final processed data have been deposited in a public database such as GEO.

☐ Confirm that you have deposited or provided access to graph files (e.g. BED files) for the called peaks.

Data access links<br>*May remain private before publication.*	N/A
Files in database submission	N/A
Genome browser session<br>(e.g. UCSC)	N/A

Methodology

Replicates	N/A
Sequencing depth	N/A

Antibodies	N/A
Peak calling parameters	N/A
Data quality	N/A
Software	N/A

Flow Cytometry

Plots

Confirm that:

- [ ] The axis labels state the marker and fluorochrome used (e.g. CD4-FITC).
- [ ] The axis scales are clearly visible. Include numbers along axes only for bottom left plot of group (a 'group' is an analysis of identical markers).
- [ ] All plots are contour plots with outliers or pseudocolor plots.
- [ ] A numerical value for number of cells or percentage (with statistics) is provided.

Methodology

Sample preparation	N/A
Instrument	N/A
Software	N/A
Cell population abundance	N/A
Gating strategy	N/A

- [ ] Tick this box to confirm that a figure exemplifying the gating strategy is provided in the Supplementary Information.

Magnetic resonance imaging

Experimental design

Design type	N/A
Design specifications	N/A
Behavioral performance measures	N/A

Acquisition

Imaging type(s)	N/A
Field strength	N/A
Sequence & imaging parameters	N/A
Area of acquisition	N/A

Diffusion MRI ☐ Used ☐ Not used

Preprocessing

Preprocessing software	N/A
Normalization	N/A
Normalization template	N/A
Noise and artifact removal	N/A

Volume censoring | N/A

Statistical modeling & inference

Model type and settings | N/A

Effect(s) tested | N/A

Specify type of analysis: ☐ Whole brain ☐ ROI-based ☐ Both

Statistic type for inference (See Eklund et al. 2016) | N/A

Correction | N/A

Models & analysis

n/a	Involved in the study
☒	☐ Functional and/or effective connectivity
☒	☐ Graph analysis
☒	☐ Multivariate modeling or predictive analysis
